# Allosteric activation of Trx1 by antagonizing nitrative modification at tyrosine 49 confers neuroprotection against ischemic stroke

**DOI:** 10.1016/j.redox.2026.104246

**Published:** 2026-06-04

**Authors:** Meizhu Guo, Huan Guo, Wenshuo Ren, Xiaonian Wang, Xing Wang, Yang Liu, Weijun Kong, Qiang Guo

**Affiliations:** a School of Traditional Chinese Medicine, Capital Medical University, Beijing, 100069, China; b Center of Basic Medical Research, Institute of Medical Innovation and Research, Peking University Third Hospital, Beijing, 100191, China

**Keywords:** Allosteric activation, Nitrotyrosine, Thioredoxin, Tetramethylpyrazine, Ischemic stroke

## Abstract

Ischemic stroke remains a leading cause of mortality and chronic disability worldwide, with limited therapeutic options. Tetramethylpyrazine (TMP) is a natural product with well-established clinical efficacy against ischemic stroke, yet its molecular target and mechanism of action remain elusive. By integrating a bifunctional photoaffinity TMP probe with stable isotope labeling by amino acids in cell culture and activity-based protein profiling (SILAC-ABPP), we identified thioredoxin 1 (Trx1) as a direct target of TMP. We demonstrate that TMP binds specifically to tyrosine 49 (Y49) on Trx1, antagonizes its nitrative modification, and functions as an allosteric activator. This binding enhances Trx1's reductase activity, strengthens its interaction with apoptosis signal-regulating kinase 1 (ASK1), and consequently suppresses the ASK1-p38/JNK signaling cascade. Genetic ablation of *Trx1* or *ASK1*, or pharmacological induction of Trx1 nitration, completely abolishes TMP-mediated neuroprotection. Our findings not only decipher the mechanistic basis for TMP's clinical efficacy but also identify Y49 of Trx1 as a druggable allosteric site, unveiling a novel anti-nitrative therapeutic strategy for ischemic stroke and related disorders involving nitrative stress.

## Highlights

• Trx1 Tyr49 is a novel druggable allosteric site.
• TMP binds Trx1 at Tyr49, blocks nitration, and allosterically activates Trx1.
• TMP enhances Trx1 reductase activity and strengthens Trx1-ASK1 binding.
• TMP suppresses ASK1-p38/JNK signaling and alleviates ischemic brain injury.
• Loss of *Trx1* or *ASK1*, or nitrative stress, abolishes TMP-mediated neuroprotection.

## Graphical abstract

**Figure:**
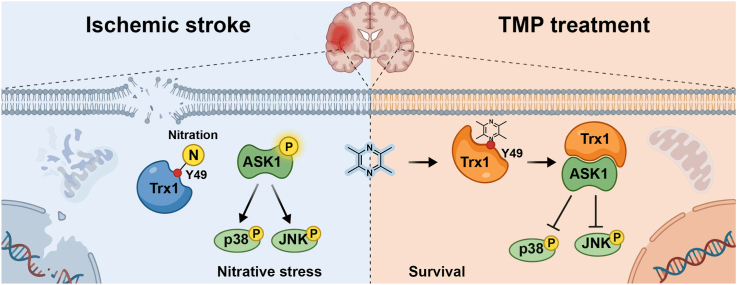

## 1 Introduction

Ischemic stroke, accounting for approximately 80% of all cerebrovascular accidents globally, is a leading cause of chronic disability and mortality [1,2]. The pathophysiology initiates from a sudden thromboembolic occlusion of cerebral arteries, resulting in oxygen-glucose deprivation, ATP depletion, and ultimately, neuronal death [3]. While current clinical management prioritizes rapid reperfusion via thrombolysis or mechanical thrombectomy, the subsequent ischemia/reperfusion (I/R) injury often aggravates the initial damage [4]. This therapeutic dilemma underscores the urgent need for neuroprotective agents capable of mitigating both the ischemic and reperfusion phases of injury. A key driver of I/R pathology is oxidative stress [5], which facilitates the reaction of nitric oxide (·NO) with superoxide (O_2_·^-^) to form peroxynitrite (ONOO^−^)—a potent oxidant that induces protein tyrosine nitration [6,7]. Beyond being a mere marker of oxidative stress, tyrosine nitration can directly impair protein function, leading to pathological inactivation, activation, or gain-of-function effects [7]. Consequently, targeting nitrative stress emerges as a promising therapeutic avenue for improving outcomes in ischemic stroke.

The thioredoxin (Trx) system is a central regulator of cellular redox homeostasis. Among its members, the cytosolic/nuclear isoform Trx1 has been extensively implicated in neurological and cerebrovascular diseases, including Alzheimer's disease [8], Parkinson's disease [9], and subarachnoid hemorrhage [10]. This ubiquitously expressed 12-kDa protein contains a canonical redox-active Cys-Gly-Pro-Cys motif [11], empowering it to function as a master regulator of oxidative stress [12,13], inflammation [14,15], and cell survival [16,17]. Through these pleiotropic functions, Trx1 plays a critical role in maintaining intracellular redox balance and safeguarding neuronal and non-neuronal cells from oxidative damage [18]. Given its pivotal role in cerebral pathophysiology, Trx1 has emerged as a compelling therapeutic target, where precise modulation of its activity holds significant promise. The functional capacity of Trx1 is dynamically regulated by various post-translational modifications (PTMs) [19]. Notably, nitrative inactivation of Trx1 has been mechanistically linked to exacerbated cell death in myocardial I/R injury [20], suggesting that analogous pathological mechanisms may operate in cerebral I/R and could be targeted by anti-nitration therapies. However, the pathological significance of Trx1 nitration in stroke remains inadequately defined. Moreover, no small-molecule agents have been identified that specifically counteract this modification, representing a critical gap in the therapeutic arsenal for brain disorders.

Tetramethylpyrazine (TMP), a bioactive alkaloid derived from the traditional Chinese herb *Ligusticum chuanxiong*, has been clinically employed in China for over five decades to treat ischemic stroke [21]. Its formulations, including TMP hydrochloride and TMP phosphate injections, are listed in China's National Reimbursement Drug List [22]. Well-documented clinical benefits include improved survival rates and functional recovery in patients [23,24]. Despite its established efficacy, the direct molecular target and the precise mechanism underlying TMP's neuroprotective effects have remained enigmatic, impeding further rational drug development and optimized clinical application.

Activity-based protein profiling (ABPP) is a powerful chemical proteomic strategy that utilizes reactive probes to covalently modify and enrich protein targets for identification [25]. However, its application is challenged by natural products like TMP, which typically engage targets through reversible, non-covalent interactions [26,27]. To overcome this limitation, photoaffinity probes can be incorporated into ABPP workflows [28,29]. These probes contain photoactive groups that, upon UV irradiation, form reactive intermediates capable of crosslinking with proximal proteins, thereby stabilizing transient interactions for subsequent identification [30,31]. Coupling ABPP with stable isotope labeling by amino acids in cell culture (SILAC) further enhances the quantitative accuracy and confidence of target identification.

In this study, we integrated a bifunctional photoaffinity TMP probe with a SILAC-ABPP platform to systematically identify Trx1 as a direct target of TMP. We demonstrate that TMP binds to tyrosine 49 (Y49), antagonizes its nitrative inactivation, and acts as an allosteric activator of Trx1. This action enhances the Trx1-apoptosis signal-regulating kinase 1 (ASK1) interaction and inhibits the downstream ASK1-p38/JNK signaling axis. Our findings elucidate the long-sought mechanistic basis for TMP's clinical utility and establish Y49 on Trx1 as a druggable allosteric site for targeting nitrative stress in stroke intervention.

## 2 Materials and methods

### 2.1 Animals and treatment

Male SD rats (280–330 g) were used in this study. All rats were maintained at a temperature of 25 ± 1 °C, with a relative humidity of 50–60%, under a 12:12 h light–dark cycle. Food and water were provided ad libitum, and the rats were housed in random groups of three per cage. Prior to the experiments, all rats were fed a commercial chow diet and acclimatized to the laboratory environment for a minimum of one week. The rats were sourced from Beijing Vital River Laboratory Animal Technology Co., Ltd. First, rats were randomly divided into seven groups (n = 25 rats/group): sham, sham + TMP (80 mg/kg), middle cerebral artery occlusion/reperfusion (MCAO/R), MCAO/R + low-dose TMP (20 mg/kg), MCAO/R + medium-dose TMP (40 mg/kg), MCAO/R + high-dose TMP (80 mg/kg), and MCAO/R + evaravone (EDA, positive drug, 10 mg/kg). TMP was administered intraperitoneally once daily for three consecutive days, with the first dose given at 1 h after reperfusion. Rats in the sham and MCAO/R groups were intraperitoneally injected with an equal volume of normal saline. Second, rats were randomly divided into five groups (n = 25 rats/group): sham, MCAO/R, MCAO/R + TMP (80 mg/kg), MCAO/R + SIN-1 (1 mg/kg), and MCAO/R + TMP (80 mg/kg) + SIN-1 (1 mg/kg). SIN-1 and TMP were administered via intraperitoneal injection once daily for three consecutive days, with the first injection administered at 0.5 h and 1 h after reperfusion, respectively. Rats in the sham and MCAO/R groups were intraperitoneally injected with an equal volume of normal saline.

### 2.2 Ethics statement

All experiments involving animals were conducted according to the ethical policies and procedures approved by the Animal Experiments and Experimental Animal Welfare Committee of Capital Medical University (Approval no. AEEI-2025-090).

### 2.3 MCAO/R model

Focal cerebral ischemia was induced by transient occlusion of the right middle cerebral artery (MCA). Briefly, the right common carotid artery (CCA), external carotid artery (ECA), and internal carotid artery (ICA) were carefully exposed and dissected from surrounding connective tissue. The CCA and ECA were permanently ligated. A small incision was made in the CCA, and a silicon-coated monofilament suture (A-5, Beijing Cinontech Co. Ltd.) was introduced into the CCA lumen and gently advanced into the ICA to occlude the origin of the MCA. After 90 min of occlusion, the filament was gently withdrawn to restore blood flow (reperfusion). Throughout the surgical procedure, body temperature was maintained at 36.5 ± 0.5 °C using a heating pad. Animals in the sham-operated group underwent the same surgical exposure and manipulation, but the filament was not inserted.

### 2.4 Neurobehavioral test

At 30 min after the final drug administration, rats were scored for neurological deficits according to a modified Longa 5-point scale [32,33]. Briefly, rats were evaluated for spontaneous locomotion, circling behavior, and postural asymmetry in an open-field environment. Motor function was assessed by suspending the rat by the tail for 30 s to monitor forelimb asymmetry (flexion of the contralateral forelimb). Gait analysis was performed on a flat surface to evaluate walking patterns. The scoring criteria were as follows: 0, no neurological deficit; 1, failure to fully extend the contralateral forepaw; 2, decreased grip strength of the contralateral forelimb when tail pulled, or circling to the contralateral side; 3, spontaneous circling or walking to the contralateral side; 4, inability to walk straight, with falling toward the paralyzed side; 5, death (excluded). Three rats were excluded from the study due to mortality following MCAO surgery.

### 2.5 2,3,5-Triphenyltetrazolium chloride (TTC) staining

Following the evaluation for neurological deficits, rats were decapitated under anesthesia, and the brain tissues were carefully removed. Three-millimeter-thick coronal sections were cut from the frontal lobe with a sharp blade. The cerebral sections were stained with TTC (0.5% w/v) at 37 °C for 30 min under light-protected conditions. Photographs were captured for subsequent ImageJ analysis of the infarction rate. The infarction rate was calculated using the formula: infarction rate (%) = (infarcted area/total brain area) × 100%.

### 2.6 HE and Nissl staining

Rats were deeply anesthetized and perfused with normal saline followed by 4% paraformaldehyde. Subsequently, the brains were removed and fixed in 4% paraformaldehyde, dehydrated through a series of ethanol solutions, and embedded in paraffin. Coronal sections of 5 μm thickness were cut for assay. The slices were then deparaffinized, rehydrated, stained with hematoxylin-eosin (HE staining) or 1% toluidine blue (Nissl staining), and imaged using a Nikon Eclipse E100 biological microscope connected to a Nikon DIGITAL SIGHT DS-U3 digital camera.

### 2.7 Cell culture

Primary hippocampal neurons were isolated from embryonic ICR mice. The dissected tissues were digested and dissociated in Hank's balanced salt solution, followed by resuspension in DMEM. Neurons were seeded onto poly-l-lysine-coated plates. After 4 h, the medium was replaced with neurobasal medium supplemented with 2% B27 and 2 mM glutamine. To suppress non-neuronal cell proliferation, cytosine arabinoside was added. All primary cultures were confirmed to be free of mycoplasma contamination prior to use. The human neuroblastoma SH-SY5Y cell line (RRID: CVCL_0019) was purchased on December 2, 2020, from the Stem Cell Bank, Chinese Academy of Sciences (Shanghai, China; Catalog Number: SCSP-5014; Batch Number: 20200112). This batch was authenticated by the provider via STR profiling and confirmed to be free of mycoplasma contamination. We also independently verified that the cells were mycoplasma-negative using a PCR-based method immediately before initiating experiments. SH-SY5Y cells were cultured in high glucose DMEM supplemented with 10% FBS, 100 U/mL penicillin, and 100 μg/mL streptomycin in a humidified incubator at 37 °C with 5% CO_2_.

### 2.8 Oxygen-glucose deprivation/reperfusion (OGD/R) model

OGD/R-induced primary neurons and SH-SY5Y cells were used to simulate I/R injury in vitro. In brief, cells were rinsed once with glucose-free Earle's balanced salt solution (116 mM NaCl, 5.4 mM KCl, 1 mM NaH_2_PO_4_, and 26.2 mM NaHCO_3_, Leagene Biotechnology, Beijing, China) and then incubated in this solution. The cells were immediately placed in a sealed chamber with an Anaero Pack (Mitsubishi, Tokyo, Japan) for 7 h to initiate OGD. Thereafter, the glucose-free Earle's balanced salt solution was replaced with fresh complete DMEM, and the cells were cultured for an additional 24 h under normoxic conditions (reperfusion). For TMP treatment, cells were treated with varying concentrations of TMP (5, 10, and 20 μM) during the OGD/R period, while untreated cells served as the control group.

### 2.9 Cell counting kit-8 (CCK-8) assay

Stock solutions of TMP or TMP-P (40 mM) were prepared in dimethyl sulfoxide (DMSO), stored at −20 °C, and diluted in cell culture medium as required. During the OGD/R period, cells were exposed to various concentrations of TMP or TMP-P. Following treatment, CCK-8 solution was added to the designated wells, and plates were incubated at 37 °C in a 5% CO_2_ atmosphere for 2 h. The absorbance of each well was then measured at 450 nm using a microplate reader. Cell viability was calculated using the following formula: cell viability (%) = (OD value of drug-treated group/OD value of normal control group) × 100%.

### 2.10 Crystal violet staining

Cells were harvested and seeded into 24-well plates. After 24 h of culture to allow adherence, drugs treatment was performed concurrently with the OGD/R induction. Subsequently, cells were fixed with 4% paraformaldehyde at room temperature for 20 min, stained with 0.1% crystal violet solution at 37 °C for 20 min, rinsed with PBS, and imaged under bright-field microscopy.

### 2.11 JC-1 staining

Log-phase cells were harvested and seeded into 24-well plates. After 24 h of culture to allow complete adhesion, cells were subjected to OGD/R and treated with TMP at concentrations of 5, 10, or 20 μM. Mitochondrial membrane potential was evaluated using the JC-1 assay. Briefly, cells were incubated with JC-1 working solution for 20 min at 37 °C, washed twice with ice-cold JC-1 staining buffer, and immediately examined under a fluorescence microscope. JC-1 monomers (depolarized mitochondria) were visualized using excitation/emission wavelengths of 514/529 nm, whereas JC-1 aggregates (polarized mitochondria) were detected at 585/590 nm. The extent of mitochondrial depolarization was quantified as the ratio of green (monomer) fluorescence intensity relative to total (green + red) fluorescence intensity.

### 2.12 Measurement of ROS

The level of intracellular ROS in SH-SY5Y cells was determined using 2′,7′-dichlorodihydrofluorescein diacetate (DCFH-DA). Under oxidative conditions, cell-permeable DCFH-DA is deacetylated by intracellular esterases to DCFH, which is subsequently oxidized by ROS to the highly fluorescent compound DCF, emitting green fluorescence. Following OGD/R treatment with or without TMP intervention, cells were incubated with DCFH-DA (10 μM) at 37 °C for 20 min in the dark. Fluorescence signals were visualized using an inverted fluorescence microscope (Nikon ECLIPSE Ti–U, Tokyo, Japan) equipped with a GFP filter. Representative images were captured, and fluorescence intensity was quantified using ImageJ software.

### 2.13 Hoechst 33258 staining

Cells were subjected to OGD/R modeling followed by treatment with TMP (5, 10, or 20 μM). After treatment, cells were washed with PBS and fixed with 4% paraformaldehyde for 20 min at room temperature. Fixed cells were washed with PBS, stained with Hoechst 33258 (10 μg/mL), and incubated for 10 min. Cells were then washed twice with PBS and visualized under a fluorescence microscope with excitation and emission wavelengths at 350 nm and 460 nm, respectively.

### 2.14 Synthetic procedures of TMP-P

2-Hydroxymethyl-3,5,6-trimethylpyrazine (337.2 mg, 2.2 mmol), 3-(3-(but-3-yn-1-yl)-3H-diazirin-3-yl)propanoic acid (142.3 mg, 0.9 mmol), 1-ethyl-(3-(3-dimethylamino)propyl)-carbodiimide hydrochloride (427.6 mg, 2.5 mmol), 4-dimethylaminopyridine (183.3 mg, 1.5 mmol), and *N*-ethyl-*N*,*N*-diisopropylamine (646.3 mg, 5 mmol) were mixed in anhydrous dichloromethane (5 mL). The mixture was stirred for 24 h at room temperature in the dark. The reaction solution was dried in vacuo and purified by silica gel column chromatography (petroleum ether/EtOAc, 4:1) to afford the product TMP-P (brown oil, 238.2 mg, 88%). ^1^H NMR (CDCl_3_, 500 MHz): *δ*_H_ 5.02 (2H, s, H-7), 2.35 (3H, s, H-8), 2.33 (3H, s, H-9), 2.32 (3H, s, H-10), 2.03 (2H, t, *J* = 7.8 Hz, H-2′), 1.65 (2H, t, *J* = 7.8 Hz, H-3′), 1.46 (2H, t, *J* = 7.5 Hz, H-5′), 1.82 (2H, m, H-6′), 1.86 (1H, m, H-8′); ^13^C NMR (CDCl_3_, 125 MHz): *δ*_C_ 148.7 (C-2), 144.2 (C-3), 148.7 (C-5), 151.1 (C-6), 64.9 (C-7), 20.2 (C-8), 21.5 (C-9), 21.2 (C-10), 171.5 (C-1′), 28.0 (C-2′), 27.7 (C-3′), 27.3 (C-4′), 31.9 (C-5′), 13.0 (C-6′), 82.3 (C-7′), 69.2 (C-8′); (+)-HRESIMS *m/z* 301.1665 [M + H]^+^ (calcd for C_16_H_21_N_4_O_2_, 301.1659).

### 2.15 SILAC-ABPP for target identification

Cell culture and metabolic labeling with SILAC: Stable isotope labeling SH-SY5Y cells were cultured in SILAC DMEM (Thermo Fisher Scientific) supplemented with 10% SILAC FBS (Thermo Fisher Scientific), 100 U/mL penicillin, 100 μg/mL streptomycin, and either heavy ([^13^C_6_,^15^N_4_]l-arginine-HCl and [^13^C_6_,^15^N_2_]l-lysine-HCl; Cambridge Isotope Laboratory) or light (l-arginine-HCl and l-lysine-HCl; Sigma-Aldrich) amino acids to replace normal arginine and lysine. Cells were cultured in labeled medium for at least seven passages to achieve >95% incorporation. Heavy-labeled cells served as the competition group (pretreated with excess free TMP), while light-labeled cells served as the experimental group (probe only), enabling quantitative comparison of protein enrichment.

In situ photocrosslinking and sample preparation: For in situ labeling, cells were grown to 80–90% confluency. Heavy-labeled cells were pretreated with TMP (200 μM) for 30 min, while light-labeled cells received DMSO. Both groups were then incubated with TMP-P (20 μM) for 4 h, and exposed to UV light (365 nm) for 10 min on ice to induce covalent crosslinking. Cells were lysed with NP-40 buffer, and heavy and light lysates were mixed at a 1:1 ratio. Click chemistry was performed by incubating samples with 100 μM biotin-PEG_3_-N_3_, 1 mM tris(2-carboxyethyl)phosphine, 100 μM tris[(1-benzyl-1H-1,2,3-triazol-4-yl)methyl]amine, and 1 mM CuSO_4_ for 1 h. Proteins were precipitated with acetone to remove unreacted reagents, washed with cold methanol, solubilized in 1.2 % SDS/PBS, and diluted 5× with PBS.

In vitro photocrosslinking and sample preparation: For in vitro labeling, cells were harvested and lysed, and protein concentrations were adjusted to 1 mg/mL. Light-labeled lysates were incubated with 20 μM TMP-P for 1 h, while heavy-labeled lysates were pretreated with 200 μM TMP for 1 h followed by incubation with 20 μM TMP-P for 1 h. Both samples were then exposed to UV light (365 nm) for 10 min on ice, mixed at a 1:1 ratio, and subjected to click chemistry and protein precipitation as described above.

LC-MS/MS analysis: The proteins were incubated with streptavidin beads for enrichment, followed by on-bead tryptic digestion. Peptide samples were analyzed on an Orbitrap Exploris 480 mass spectrometer coupled to an Easy n-LC 1200 HPLC system. Peptides were loaded onto a trap column (100 μm id × 2 cm, packed with 5 μm Reprosil-Pur C18 AQ) and separated on an analytical column (75 μm id × 20 cm, packed with 1.9 μm Reprosil-Pur C18 AQ) using a 120 min linear gradient from 4% to 95% buffer B (A: 0.1% formic acid in water; B: 80% acetonitrile with 0.1% formic acid) at a flow rate of 300 nL/min. MS data were acquired in data-dependent acquisition mode (*m/z* 350–1500) with a resolution of 120,000, automatic gain control (AGC) target of 3.00E+06, and maximum injection time of 22 ms. MS/MS scans were performed at a resolution of 30,000 with an AGC target of 7.50E+04, maximum injection time of 54 ms, and normalized collision energy of 28%.

The mass spectrometry proteomics data have been deposited to the ProteomeXchange Consortium via the PRIDE partner repository with the dataset identifier PXD077143.

### 2.16 Recombinant human Trx1 (rhTrx1) protein expression and purification

Trx1 with an N-terminal GST tag was cloned into the pGEX-6P-1 vector and expressed in *E. coli* BL21 (DE3) cells. Bacteria were cultured at 37 °C for 4 h, followed by induction with 0.5 mM isopropyl β-d-1-thiogalactopyranoside and further cultured at 20 °C for 12 h. The protein was purified using glutathione agarose and subsequently cleaved with PreScission protease to remove the GST tag. Protein purity was analyzed by SDS-PAGE and Coomassie blue staining.

### 2.17 Microscale thermophoresis (MST) measurement

Purified tag-free fatty acid-binding protein 5 (FABP5) and Trx1 proteins (200 nM each) were buffer-exchanged into PBS and labeled according to the protocol of the Protein labelling kit (Nanotemper Technologies, L001). Labeled FABP5 or Trx1 were mixed with various concentrations of TMP in reaction buffer (20 mM PBS, pH 7.4, 150 mM NaCl). After a 5 min incubation at room temperature, the mixture was loaded into capillaries for measurement. MST experiment was conducted using a Monolith NT.115 instrument (NanoTemper Technologies) with 20% LED/excitation power and 40% MST power. Data were analyzed using NanoTemper analysis software, and the dissociation constant (*K*_D_) was determined.

### 2.18 Co-localization study

Following OGD/R modeling and treatment with 20 μM TMP-P, cells were exposed to 365 nm UV light for 10 min on ice, fixed with 4% paraformaldehyde at room temperature for 20 min, and permeabilized with 0.5% Triton X-100. Cells were then incubated with a freshly prepared click chemistry cocktail (50 μM TAMRA-PEG_3_-N_3_, 1 mM TCEP, 100 μM TBTA, and 1 mM CuSO_4_) with gentle shaking for 2 h at room temperature. After blocking with 5% BSA, cells were incubated with Trx1 primary antibody (1:200) at 4 °C overnight, followed by Alexa Fluor 488-conjugated secondary antibody (1:500) for 1 h, and then incubated with DAPI for 10 min at room temperature. Images were acquired using a Leica TCS SP8 STED confocal fluorescence microscopy. Colocalization coefficients were calculated using ImageJ software.

### 2.19 Pull-down analysis

Probe binding and photocrosslinking: rhTrx1 or SH-SY5Y cell lysates were pre-incubated with or without TMP (50, 100, or 200 μM) for 1 h, followed by addition of TMP-P (20 μM) or an equivalent volume of DMSO as a control. After further incubation for 1 h, samples were irradiated with 365 nm UV light on ice for 10 min to induce covalent crosslinking.

Biotin conjugation and protein precipitation: Following photocrosslinking, click chemistry conjugation to biotin-azide was performed as described in section 2.15. Reaction mixtures were centrifuged, and the resulting pellets were washed with ice-cold methanol.

Protein solubilization and bead capture: Washed pellets were dissolved in PBS containing 1.2% SDS, after which the SDS concentration was diluted to 0.2% with PBS. Samples were incubated with 50 μL of streptavidin-coated beads for 3 h at room temperature with gentle rotation.

Washing and elution for analysis: The beads were washed three times with PBS containing 0.2% SDS, and bound proteins were eluted by heating in 2× SDS-PAGE loading buffer at 98 °C for 10 min and analyzed by western blotting using the indicated antibodies.

### 2.20 Cellular thermal shift assays (CETSA) assay

SH-SY5Y cells were treated with or without 20 μM TMP for 2 h. After treatment, cells were collected, resuspended in PBS, and equally distributed into 200 μL PCR tubes. Tubes were heated at 37, 40, 43, 46, 49, 52, 55, 58, 61, or 64 °C for 3 min. Cells were then resuspended in kinase buffer (25 mM Tris-HCl, pH 7.5, 5 mM β-glycerophosphate, 2 mM DTT, 0.1 mM Na_3_VO_4_, 10 mM MgCl_2_) and subjected to three cycles of freeze-thawing in liquid nitrogen. The resulting cell lysates were collected for subsequent western blotting analysis.

### 2.21 Drug affinity responsive target stability (DARTS) assay

SH-SY5Y cells were lysed in NP-40 lysis buffer supplemented with protease inhibitor cocktail. Lysates were centrifuged at 18,000 × g for 10 min at 4 °C to remove insoluble debris, and the supernatant was collected and diluted with TNC buffer (50 mM Tris-HCl, pH 8.0, 50 mM NaCl, 10 mM CaCl_2_). Diluted lysates were incubated with TMP (5, 10, or 20 μM) or an equivalent volume of vehicle for 1 h at room temperature with gentle rotation. Proteolytic digestion was initiated by adding pronase (Roche Diagnostics) to a final concentration of 2 μg/mL, and samples were incubated at room temperature for exactly 20 min. The digestion was terminated by adding 5× SDS-PAGE loading buffer and immediately heating at 98 °C for 10 min. Samples were then subjected to western blot analysis using the indicated antibodies.

### 2.22 Lentivirus packaging and transduction

The *Trx1*-knockout (Trx1-KO) and *ASK1*-knockout (ASK1-KO) SH-SY5Y cell lines were generated via CRISPR/Cas9-mediated genome editing. To knock out *Trx1* or *ASK1* in cells, the U6 promoter-dependent lentivirus vector lentiCRISPR v2 was used. The target sequence for *Trx1* was 5′-GCAGGAAGCCTTGGACGCTGC-3′. The target sequence for *ASK1* was 5′-GGTAAAACAAGGACGGCTGC-3′. To generate SH-SY5Y cells stably overexpressing HA-Trx1 or HA-Trx1^Y49A^, the corresponding cDNAs were cloned into the pCDH-CMV-MCS-EF1-copGFP-T2A-Puro vector. All engineered lentiviral vectors were packaged into HEK293T cells to produce lentivirus. An empty vector served as a control. The virus-containing supernatant was filtered through a 0.45 μm filter prior to transduction. For lentivirus infection, SH-SY5Y cells were seeded in 6-well plates and incubated for 24 h. Cells were then infected with knockout or overexpression lentivirus, or control lentivirus, in the presence of 2 μg/mL polybrene. After 24 h, the culture medium was replaced with fresh DMEM complete medium for another 48 h. Stable cell lines were selected using puromycin (2 μg/mL). Protein knockout or overexpression was evaluated by western blot. SH-SY5Y cells infected with empty lentiviral vector served as a control.

### 2.23 Identification of binding sites

rhTrx1 (0.83 μM) was incubated with TMP-P (20 μM) for 1 h, followed by UV irradiation (365 nm) for 10 min on ice to induce covalent crosslinking. The protein was resolved by 15% SDS-PAGE and visualized by Coomassie blue staining. Protein bands corresponding to Trx1 were excised, destained, and subjected to in-gel tryptic digestion. Extracted peptides were analyzed using an Orbitrap Fusion Lumos mass spectrometer coupled to an EASY-nLC 1200 system. Peptide samples (2 μL) were loaded onto a trap column (5 μm C18) at 5 μL/min, then separated on an analytical column (150 μm × 22 cm, 1.9 μm C18) at 500 nL/min with a linear gradient of 7–40% solvent B (0.1% formic acid in acetonitrile) over 70 min, followed by a wash at 99% B.

The Orbitrap Fusion Lumos mass spectrometer, equipped with a nanoelectrospray ion source (spray voltage: +2200 V; ion transfer tube temperature: 320 °C) was operated in data-dependent acquisition mode with a cycle time of 3 s. Full-scan MS spectra (*m/z* 350–1550) were acquired in the Orbitrap analyzer at a resolution of 120,000. Precursor ions (charge states of 2–6, intensity >5000) were selected for MS/MS analysis, isolated with a quadrupole isolation window of 1.6 *m/z* and fragmented by HCD with a collision energy of 32%. MS/MS spectra were acquired in the ion trap analyzer in rapid scan mode. Dynamic exclusion was enabled with a single count and a duration of 15 s.

Mass spectrometric data were analyzed using Proteome Discoverer software against the UniProt human protein database. Search parameters included: enzyme, trypsin; maximum missed cleavages, 2; variable modifications, oct-7-ynoic acid (+138.0681 Da) and intact photoactivated tag (+272.1525 Da); precursor mass tolerance, ±5 ppm; fragment mass tolerance, 0.6 Da; false discovery rate threshold, 0.01.

The mass spectrometry proteomics data have been deposited to the ProteomeXchange Consortium via the PRIDE partner repository with the dataset identifier PXD077200.

### 2.24 Trx1 activity assay

After modeling, cells were treated with TMP at concentrations of 5, 10, or 20 μM, and animals were administered TMP at doses of 20, 40, or 80 mg/kg via intraperitoneal injection. SH-SY5Y cells or rat brain tissues were harvested, and total proteins were extracted. Protein concentration was determined using a BCA kit and adjusted to 1 mg/mL. Trx1 activity in total protein lysates was evaluated using a thioredoxin activity fluorescent assay kit (Cayman Chemical, MI, USA). Briefly, 20 μg of total protein or various amounts of rhTrx1 were incubated with recombinant thioredoxin reductase and reduced β-NADPH at 37 °C for 30 min, followed by addition of eosin-labeled insulin and further incubation for 5 min. Fluorescence emission at 560 nm (excitation 520 nm) was recorded for 60 min at room temperature using a fluorescent plate reader. Activity was calculated based on the increase in fluorescence intensity within the linear range of the reaction.

### 2.25 Immunoprecipitation (IP) and co-IP

Cells were collected and lysed in ice-cold NP-40 lysis buffer for 45 min on ice. Lysates were clarified by centrifugation at 20,000 × g for 15 min at 4 °C, and the supernatants were collected. Protein concentrations were determined by BCA assay, and equal amounts of protein were adjusted across samples using fresh lysis buffer. Equalized lysates were incubated overnight at 4 °C with antibodies against Trx1, ASK1, or HA. Subsequently, 50 μL of Protein A/G beads were added to each sample and incubated for an additional 4 h at 4 °C. Non-specifically bound proteins were removed by washing three times with PBS containing 0.1% Tween-20. Immunoprecipitated complexes were eluted by boiling in 2× protein loading buffer at 98 °C for 10 min. The levels of 3-nitrotyrosine, ASK1, or Trx1 were analyzed by western blotting.

### 2.26 Nitrotyrosine site mapping by LC-MS/MS

SH-SY5Y cells were subjected to OGD/R treatment, and Trx1 was immunoprecipitated using an anti-Trx1 antibody. Separately, rhTrx1 (1 μM) was incubated with SIN-1 (10 μM) at 37 °C for 30 min. Proteins were resolved by 15% SDS-PAGE and visualized by Coomassie blue staining. Gel bands corresponding to Trx1 were excised, destained, and subjected to in-gel digestion. Briefly, gel pieces were destained, dehydrated with acetonitrile, reduced with 20 mM DTT at 37 °C for 4 h, and alkylated with 50 mM iodoacetamide at room temperature for 30 min in the dark. Digestion was performed overnight at 37 °C with an enzyme-to-protein ratio of approximately 1:100 using either trypsin or chymotrypsin. The resulting peptides were extracted from the gel pieces, dried under vacuum, and reconstituted in 0.1% formic acid for LC-MS/MS analysis.

Extracted peptides were separated using a Vanquish Neo nanoflow LC system coupled to an Orbitrap Exploris 480 mass spectrometer. Samples were directly autosampled and loaded onto a trap column (300 μm i.d. × 0.5 cm) packed with 5 μm C18 reversed-phase material. Peptide mixtures were separated on an analytical column (100 μm i.d. × 40 cm) packed with 3 μm C18 reversed-phase material and eluted with the following gradient: 0–1 min, 8% B; 1–43 min, 8–35% B; 43–63 min, 35–55% B; 63–64 min, 55–100% B; 64–66 min, 100% B; 66–70 min, 100% B (solvent A: 0.1% formic acid in water; solvent B: 0.1% formic acid in acetonitrile). The flow rate was 0.6 μL/min. The column temperature in the autosampler was maintained at 7 °C.

The Orbitrap Exploris 480 mass spectrometer, equipped with a nanoelectrospray ion source, was operated in data-dependent positive ion mode. The spray voltage was set to 2.2 kV, and the ion transfer tube temperature was maintained at 320 °C. FAIMS was enabled with compensation voltages of −45 V and −65 V. Full-scan MS spectra (*m*/*z* 350–1500) were acquired in the Orbitrap analyzer in profile mode at a resolution of 60,000 (at *m*/*z* 200). The AGC target was set to 300% (3.00E+06 ions), and the maximum injection time was set to auto. The RF lens was set to 50%. Data-dependent acquisition was performed with a cycle time of 1 s. The most abundant precursor ions with charge states 2–6 from each MS scan were selected for MS/MS fragmentation using an isolation window of 1.6 *m*/*z*. HCD was applied with a normalized collision energy of 30%. MS/MS spectra were acquired in centroid mode in the Orbitrap analyzer at a resolution of 15,000 (at *m*/*z* 200). The AGC target for MS/MS was set to 100% (1.00E+06 ions) with a custom maximum injection time of 22 ms. Dynamic exclusion was enabled with an exclusion duration of 40 s to avoid repeated sequencing of abundant precursors. A minimum intensity threshold of 8000 was required for precursor selection.

Mass spectrometric data were analyzed using Proteome Discoverer software with the SEQUEST search engine. Search parameters were as follows: taxonomy, *Homo sapiens*; enzyme, trypsin or chymotrypsin; maximum missed cleavage sites, 2; variable modifications included tyrosine nitration (+44.9851 Da), cysteine glutathionylation (+305.0682 Da), and cysteine *S*-nitrosylation (+28.9902 Da); precursor mass tolerance, 10 ppm; fragment mass tolerance, 0.02 Da; false discovery rate threshold, 0.01.

The mass spectrometry proteomics data have been deposited to the ProteomeXchange Consortium via the PRIDE partner repository with the dataset identifier PXD077236.

### 2.27 Measurement of mitochondrial membrane potential using tetramethylrhodamine methyl ester (TMRM)

Mitochondrial membrane potential was measured using TMRM. SH-SY5Y cells were seeded in 24-well plates and subjected to OGD/R treatment with or without TMP intervention. After treatment, cells were incubated with 5 μM TMRM in fresh medium for 30 min at 37 °C in the dark. After incubation, cells were washed twice with warm PBS to remove unincorporated dye and then maintained in dye-free medium during image acquisition under a fluorescence microscope.

### 2.28 Tryptophan fluorescence quenching study

rhTrx1 protein (30 μM) was titrated with increasing concentrations of TMP (0–200 μM) in a 96-well quartz microplate. Fluorescence intensities were measured using a FlexStation 3 microplate reader (Molecular Devices, CA, USA) with excitation at 280 nm and emission recorded from 300 to 500 nm at 1 nm increment. Fluorescence intensities were corrected by buffer contribution.

### 2.29 Circular dichroism (CD) spectroscopy and BeStSel analysis

Conformational changes in Trx1 were monitored using a Jasco J-810 spectrophotometer (Jasco, Tokyo, Japan). A quartz cuvette with a path length of 1 mm was used to record CD spectra at a temperature of 298 K for 10 μM rhTrx1 in the presence of varying concentrations of TMP. Spectra were acquired over a wavelength range of 190–250 nm with a data interval of 0.1 nm and a scanning speed of 100 nm/min. Each spectrum represented an average of three successive scans. To eliminate contributions from TMP, a blank containing the same concentration of TMP (without rhTrx1) was subtracted from each spectrum. Secondary structure content was estimated using the BeStSel analysis server (http://bestsel.elte.hu).

### 2.30 Molecular docking and molecular dynamics (MD) simulation

Molecular docking was performed to investigate the binding mode between TMP and Trx1. The three-dimensional structure of TMP was obtained from the PubChem database in SDF format. The crystal structure of human Trx1 was retrieved from the RCSB Protein Data Bank (PDB: 1ERU). Hydrogen atoms were added, and crystallographic water molecules were removed. The AMBER7 F99 force field was used to optimize the Trx1 structure with default parameters in SYBYL-X 2.0 software package. TMP was energy-minimized using the Tripos force field and conjugate gradient method [34]. Docking simulations were conducted using SYBYL-X 2.0 to identify the TMP binding site on Trx1 and to characterize specific interactions. Resulting docking poses were visualized and analyzed with PyMOL.

MD simulations were employed to further characterize the dynamics and binding energetics of the TMP-Trx1 complex. The initial coordinates were taken from the best docking pose. Each system was energy-minimized and equilibrated following established protocols [35,36] before production runs. A 40 ns production simulation was conducted under isothermal-isobaric conditions with periodic boundary constraints. The integration time step was set to 2 fs, and bonds involving hydrogen atoms were constrained using the SHAKE algorithm. Long-range electrostatic interactions were treated with the particle-mesh Ewald method, and a cutoff of 10.0 Å was applied for short-range non-bonded interactions.

### 2.31 Western blot analysis

Cells or tissues were lysed using RIPA or NP-40 lysis buffer supplemented with protease inhibitor cocktail. Lysates were then centrifuged at 20,000 × g for 15 min at 4 °C. Supernatants were collected, and protein concentration determination using a BCA protein assay kit. Proteins were separated by 8–15% SDS-PAGE and transferred to PVDF membranes. Membranes were blocked with 5% nonfat milk in 1× Tris-buffered saline containing Tween-20 for 1 h at room temperature, followed by overnight incubation with primary antibodies at 4 °C. After washing, membranes were incubated with HRP-conjugated secondary antibodies for 1 h at room temperature. Signal detection was performed using an ECL Super Kit. Band intensities were quantified using ImageJ software. β-actin was used as a loading control, and the relative amount of each protein from untreated cells was set to 1.

### 2.32 Statistical analysis

Data were presented as mean ± SD and analyzed using GraphPad Prism version 9.5.1 (GraphPad Software, La Jolla, CA, USA). Statistical comparisons between two groups were performed using two tailed Student's t-test. Multiple group comparisons were performed using one-way ANOVA followed by Bonferroni's post hoc test for multiple comparisons. A value of *P* < 0.05 was considered statistically significant.

## 3 Results

### 3.1 TMP alleviates cerebral I/R injury in a rat MCAO/R model

**Fig. 1:**
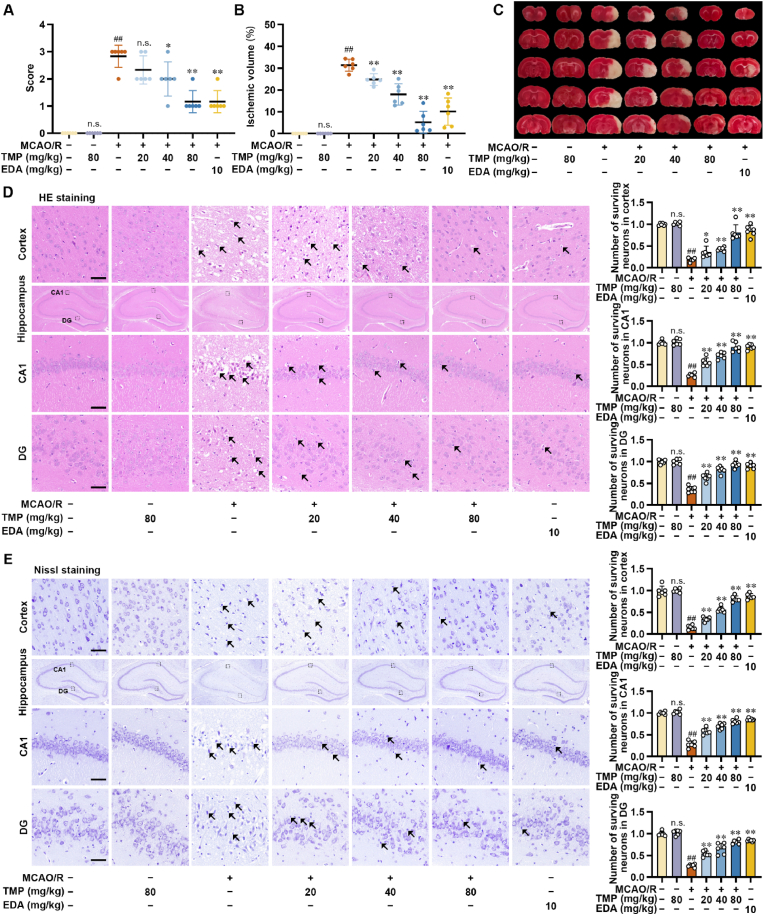
TMP alleviates cerebral I/R injury in a rat MCAO/R model. (A) Neurological deficit scores at day 3 (n = 6). (B) TMP reduced ischemic volume induced by MCAO/R (n = 6). (C) Representative photographs of TTC-stained coronal sections (n = 6). (D) HE staining showing morphological neuronal changes and the number of intact cells in the cortex and hippocampus after MCAO/R injury (scale bar: 50 μm, n = 6). Arrows indicate damaged neurons. (E) Nissl staining showing morphological neuronal changes and the number of intact cells in the cortex and hippocampal region after MCAO/R injury (scale bar: 50 μm, n = 6). Arrows indicate damaged neurons. Data were presented as mean ± SD. n.s., *P* > 0.05, ^##^*P* < 0.01 compared with sham group; n.s., *P* > 0.05, ∗*P* < 0.05, ∗∗*P* < 0.01 compared with MCAO/R group.

To investigate the neuroprotective effects of TMP against cerebral I/R injury, we established an MCAO/R rat model. Compared with sham-operated controls, rats subjected to MCAO/R exhibited pronounced neurological impairments, including hemiplegia and depressed consciousness, whereas TMP administration significantly ameliorated these deficits. Notably, sham-operated rats treated with the highest dose of TMP displayed no neurological abnormalities (Fig. 1A). Consistently, TTC staining revealed extensive cerebral infarction in MCAO/R model rats, while TMP treatment markedly reduced the infarct volume. No ischemic lesions were detected in either sham-operated group (Fig. 1B and C). Histopathological examination by HE staining further demonstrated that TMP substantially alleviated MCAO/R-induced cytoarchitectural disruption in both the cortex and hippocampus, including aberrant neuronal arrangement, diffuse vacuolization, and interstitial edema. Brain sections from sham-operated rats treated with TMP exhibited preserved histoarchitecture with no evidence of necrosis or tissue edema (Fig. 1D). Moreover, Nissl staining indicated that TMP intervention significantly mitigated MCAO/R-induced neurodegeneration, as reflected by reduced cytoplasmic shrinkage, nuclear condensation, and loss of Nissl bodies. Neurons in the sham-operated rats treated with TMP retained abundant and well-organized Nissl bodies, supporting that TMP exerts neuroprotection by targeting pathological alterations without disrupting normal neuronal integrity (Fig. 1E). Collectively, these in vivo results demonstrate that TMP confers robust neuroprotection against MCAO/R-induced cerebral injury.

### 3.2 TMP attenuates OGD/R-induced neuronal injury and mitochondrial dysfunction

**Fig. 2:**
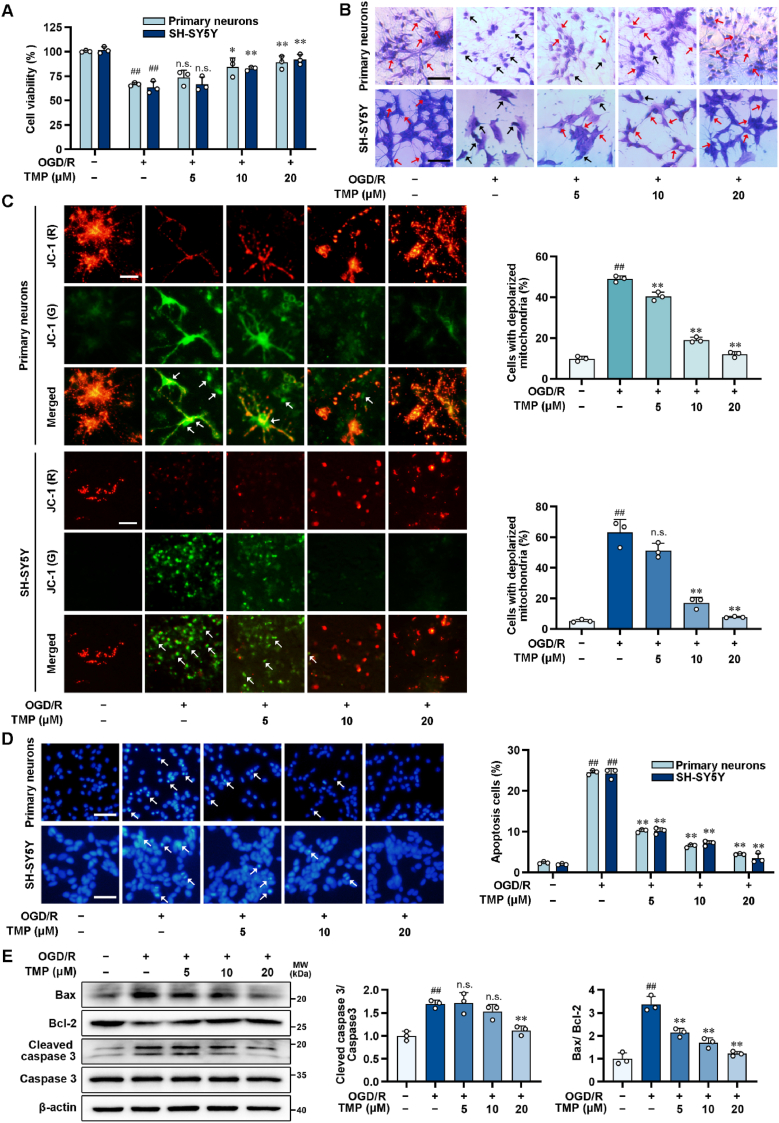
TMP attenuates OGD/R-induced neuronal injury and mitochondrial dysfunction. (A) TMP inhibited OGD/R-induced decrease in viability in primary neurons and SH-SY5Y cells (n = 3). (B) Crystal violet staining of primary neurons and SH-SY5Y cells (scale bar: 50 μm). Red arrows indicate normal cells, black arrows indicate damaged cells. (C) TMP inhibited OGD/R-induced mitochondrial depolarization in primary neurons and SH-SY5Y cells, as determined by JC-1 staining assay (scale bar: 50 μm, n = 3). Arrows indicate cells with depolarized mitochondria. (D) TMP inhibited OGD/R-induced chromatin condensation and nuclear shrinkage in primary neurons and SH-SY5Y cells, as determined by Hoechst 33258 staining assay (scale bar: 50 μm, n = 3). Arrows indicate brightly stained condensed nuclei for apoptosis. (E) Western blotting of Bax, Bcl-2, Cleaved caspase 3, Caspase 3 levels and their quantitation (n = 3). Data were presented as mean ± SD. ^##^*P* < 0.01 compared with control group; n.s., *P* > 0.05, ∗*P* < 0.05, ∗∗*P* < 0.01 compared with OGD/R group.

We next evaluated the neuroprotective effects of TMP against OGD/R-induced injury in primary neurons and SH-SY5Y cells. OGD/R exposure significantly reduced cell viability, whereas TMP treatment reversed this effect in a concentration-dependent manner. Specifically, TMP at 5, 10, and 20 μM conferred neuroprotection rates of 19.90%, 53.01%, and 66.98% in primary neurons, and 8.95%, 52.58%, and 77.96% in SH-SY5Y cells, respectively (Fig. 2A). Crystal violet staining further revealed that TMP treatment preserved synaptic integrity following OGD/R insult (Fig. 2B). Given the critical involvement of mitochondrial dysfunction in the pathogenesis of I/R injury, we assessed mitochondrial membrane potential using JC-1 staining. OGD/R-induced mitochondrial depolarization was markedly attenuated by TMP treatment (Fig. 2C). To assess intracellular ROS levels, we performed DCFH-DA staining. Increased ROS levels were detected in the OGD/R group than in the control group, whereas TMP treatment (5, 10, and 20 μM) significantly reduced ROS levels (Supplementary Fig. S1). Nuclear morphological assessment via Hoechst 33258 staining further demonstrated that TMP alleviated OGD/R-induced chromatin condensation and nuclear shrinkage (Fig. 2D). Immunoblotting analysis revealed that OGD/R upregulated cleaved caspase-3 and Bax while downregulating Bcl-2, and these molecular alterations were reversed by TMP intervention (Fig. 2E). Together, these findings indicate that TMP protects against OGD/R-induced neuronal injury by preserving mitochondrial function and mitigating cellular damage.

### 3.3 SILAC-ABPP identifies Trx1 as a direct target of TMP

**Fig. 3:**
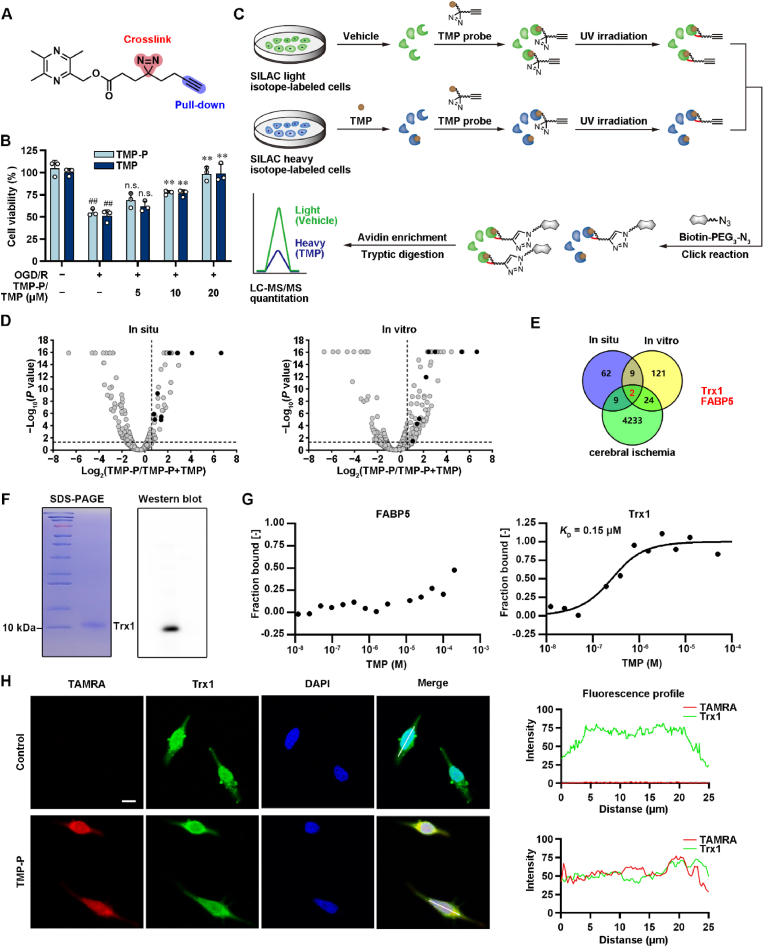
SILAC-ABPP identifies Trx1 as a direct target of TMP. (A) Structure of TMP-P. (B) Cell viability of OGD/R-injured SH-SY5Y cells treated with TMP or TMP-P determined by CCK-8 assay (n = 3). (C) Schematic illustration of target identification using the SILAC-ABPP strategy. (D) Volcano plot of quantitative proteomics (n = 3). The highly reliable proteins identified in both analyses for in situ and in vitro are prominently highlighted in black. (E) Venn diagram showing the number of potential targets identified in each set of experiments and cerebral ischemia-related proteins retrieved from the human gene database. The red intersection is the common proteins Trx1 and FABP5. (F) Coomassie blue staining and immunoblotting analysis of rhTrx1. (G) MST result of the affinity between TMP and potential target proteins. The equilibrium dissociation constant *K*_D_ of TMP and Trx1 is 0.15 μM. (H) Immunofluorescence staining of Trx1 proteins (green) and TMP-P clicked -conjugated to a TAMRA dye (red) in SH-SY5Y cells (scale bar: 10 μm). The fluorescence profiling of TAMRA and Trx1 was analyzed using ImageJ. Data were presented as mean ± SD. ^##^*P* < 0.01 compared with control group; n.s., *P* > 0.05, ∗∗*P* < 0.01 compared with OGD/R group.

To identify the cellular target of TMP, a bifunctional photoaffinity probe (TMP-P) was synthesized by conjugating a compact alkyl-diazirine-alkyne linker to the hydroxyl group of a TMP derivative via an ester bond (Fig. 3A, Supplementary Fig. S2). The structure and purity (>95%) of TMP-P were confirmed by HRESIMS, 1D and 2D-NMR, and HPLC analysis (Supplementary Fig. S3–9). The alkyl-diazirine moiety serves as a photoreactive group that, upon UV irradiation, generates a carbene intermediate capable of covalently crosslinking to interacting proteins, thereby enabling the capture of transient drug–target interactions in their native cellular environment. The terminal alkyne handle permits subsequent bioorthogonal conjugation to biotin-azide via click chemistry, facilitating streptavidin-based enrichment and LC-MS/MS identification of labeled proteins (Fig. 3A). Importantly, TMP-P retained neuroprotective activity comparable to that of unmodified TMP, as evidenced by its ability to protect SH-SY5Y cells against OGD/R-induced injury (Fig. 3B). Given the inherent potential for nonspecific labeling associated with photoaffinity probes [37], we employed a competitive SILAC-ABPP strategy in SH-SY5Y cells to discriminate genuine interactions from background [[38], [39], [40]]. Briefly, cells cultured in light isotope medium were treated with TMP-P alone, whereas those in heavy medium were pre-incubated with excess free TMP prior to TMP-P labeling as a competitive control (Fig. 3C). Proteins were considered specifically enriched only if they met two rigorous criteria: a statistically significant enrichment in the probe-only sample relative to the competition sample (*P* < 0.05) and a SILAC light/heavy ratio exceeding 1.5. Applying these filters, we identified 82 high-confidence candidate proteins from in situ labeling experiments and 156 from in vitro incubations, as depicted in the volcano plot (Fig. 3D) and listed comprehensively in Supplementary Tables S1 and S2. Intersection of these candidates with cerebral ischemia-associated proteins curated from human gene database highlighted two high-priority targets: FABP5 and Trx1 (Fig. 3E).

For orthogonal validation, recombinant expression systems were established. GST-tagged proteins were expressed, affinity purified, and cleaved to obtain tag-free target proteins (Fig. 3F). MST assays quantitatively demonstrated strong binding between TMP and Trx1 (*K*_D_ = 0.15 μM), whereas no significant interaction was detected between TMP and FABP5 (Fig. 3G). Confocal microscopy confirmed the spatial correlation, revealing near-perfect colocalization of TMP-P (red) with endogenous Trx1 (green), with Pearson's correlation and overlap coefficients of 0.983 and 0.996, respectively (Fig. 3H). These results establish Trx1 as a direct cellular target of TMP.

### 3.4 Trx1 is essential for TMP-mediated neuroprotection

**Fig. 4:**
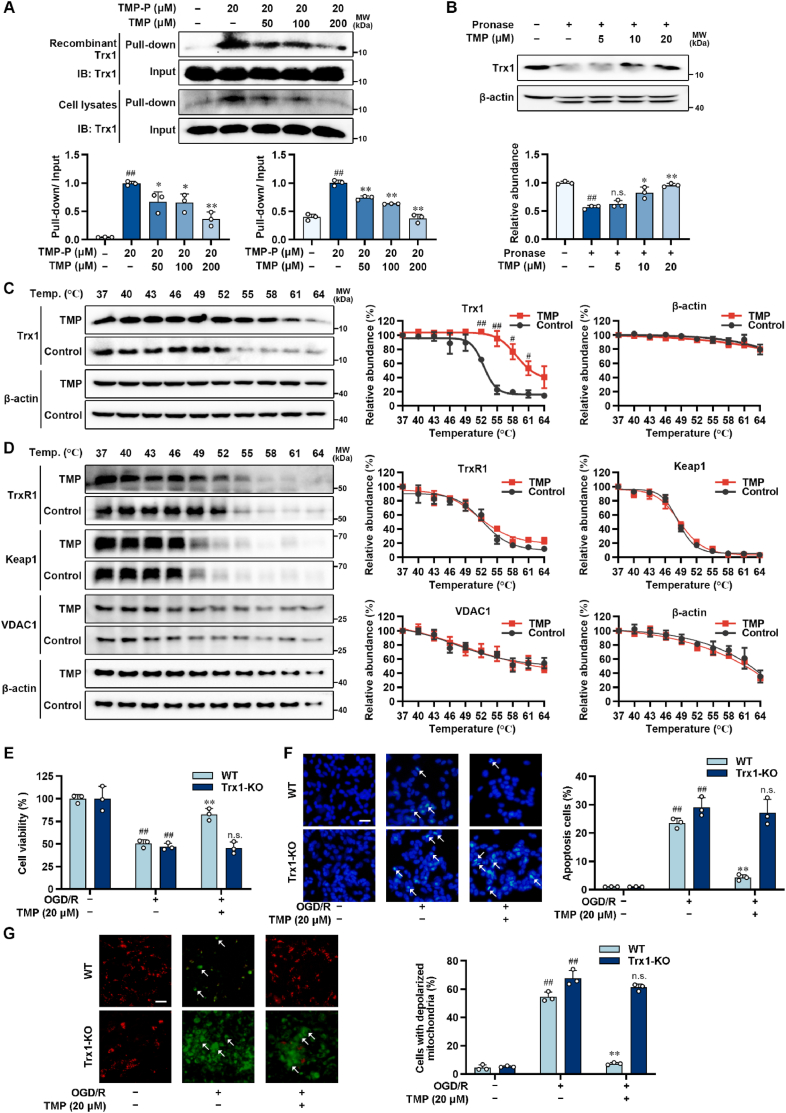
Trx1 is essential for TMP-mediated neuroprotection. (A) Pull-down analysis and statistic quantification of binding between TMP and rhTrx1 or endogenous Trx1 in SH-SY5Y cells (n = 3). (B) DARTS analysis and statistic quantification of binding between TMP and Trx1 (n = 3). (C) CETSA analysis and statistic quantification of binding between TMP and Trx1 (n = 3). (D) CETSA show that TMP does not stabilize the proteins TrxR1, Keap1, or VDAC1 in SH-SY5Y cells (n = 3). (E) Cell viability of WT and Trx1-KO SH-SY5Y cells determined by CCK-8 assay (n = 3). WT, wide type. (F) Hoechst 33258 staining of WT and Trx1-KO SH-SY5Y cells (scale bar: 50 μm, n = 3). (G) JC-1 staining of WT and Trx1-KO SH-SY5Y cells (scale bar: 50 μm, n = 3). Data were presented as mean ± SD. ^#^*P* < 0.05, ^##^*P* < 0.01 compared with control group; n.s., *P* > 0.05, ∗*P* < 0.05, ∗∗*P* < 0.01 compared with TMP-P, pronase or OGD/R group.

We further validated the specificity of the TMP-Trx1 interaction using competitive pull-down assays. Unlabeled TMP dose-dependently reduced the enrichment of TMP-P with both recombinant and endogenous Trx1. Specifically, at TMP concentrations of 50, 100, and 200 μM, the inhibition rates of Trx1 binding were 32.71%, 34.33%, and 63.50% for recombinant Trx1, and 26.23%, 36.82%, and 62.47% for endogenous Trx1 in cell lysates, respectively (Fig. 4A). DARTS analysis showed that pronase treatment led to partial degradation of both β-actin (appearing as a double-band pattern) and Trx1 (reduced band intensity). Notably, TMP treatment selectively protected Trx1 from proteolysis without affecting the degradation profile of β-actin (Fig. 4B), confirming specific target engagement in living cells. This finding was further corroborated by CETSA assays, which revealed that TMP significantly enhanced the thermal stability of Trx1 in SH-SY5Y cells, whereas the melting profile of β-actin remained unchanged (Fig. 4C).

To evaluate the binding specificity of TMP for Trx1 relative to other potential off-targets, we assessed its interaction with thioredoxin reductase 1 (TrxR1), the immediate upstream regulator responsible for maintaining Trx1 in its active, reduced state [41,42]; Kelch-like ECH-associated protein 1 (Keap1), the primary cytoplasmic repressor of the antioxidant master regulator Nrf2 [43]; and voltage-dependent anion channel 1 (VDAC1), a redox-sensitive outer mitochondrial membrane pore that integrates metabolic flux and apoptosis signaling [44,45]. In stark contrast to the pronounced thermal stabilization observed for Trx1, TMP treatment did not induce any significant shift in the melting curves of TrxR1, Keap1, or VDAC1 in SH-SY5Y cells (Fig. 4D). Together, these results robustly confirm that TMP is a direct and specific binder of Trx1.

To determine whether Trx1 is functionally required for TMP-mediated neuroprotection, we generated Trx1-KO SH-SY5Y cells using CRISPR/Cas9. Ablation of *Trx1* completely abolished the ability of TMP to restore cell viability following OGD/R exposure (Fig. 4E). Consistently, TMP failed to attenuate OGD/R-induced chromatin condensation, nuclear shrinkage, or mitochondrial depolarization in Trx1-KO cells (Fig. 4F and G). These data conclusively demonstrate that Trx1 is indispensable for the neuroprotective effects of TMP.

### 3.5 TMP allosterically activates Trx1 by inhibiting nitrative modification at Y49

**Fig. 5:**
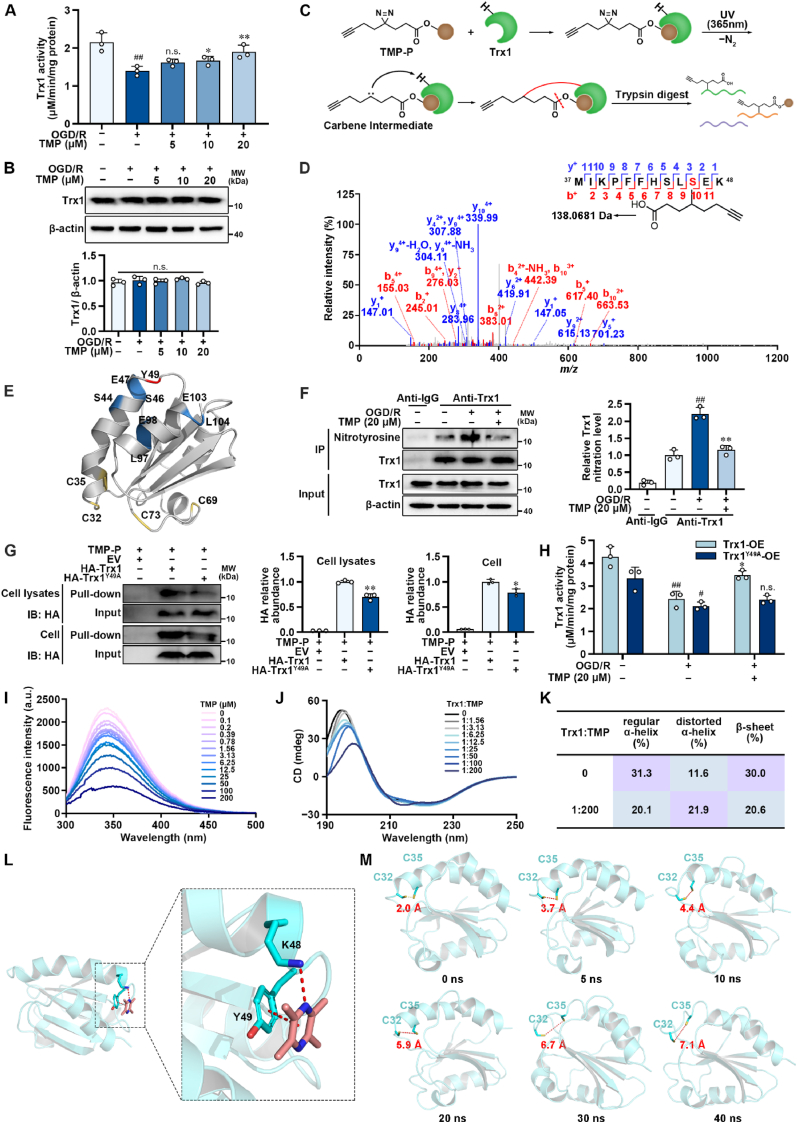
TMP allosterically activates Trx1 by inhibiting nitrative modification at Y49. (A) Trx1 activity attenuated after OGD/R treatment and restored by TMP in SH-SY5Y cells (n = 3). (B) Western blot analysis of Trx1 levels and their quantification (n = 3). (C) Schematic mechanism of diazirine-based photoaffinity crosslinking via carbene insertion into C–H bonds. (D) Representative MS/MS spectrum for the identification of TMP-*P*-labeled Trx1 peptide containing the putative cross-linked residue S46 by photo-cross-linking, ^37^MIKPFFHSLSEK^48^. (E) Cartoon representation of Trx1 showing probe modified residues (S44, S46, E47, L97, E98, E103, and L104) and PTM sites (C32, C35, Y49, C69, and C73). (F) Trx1 nitration determined by IP assays in SH-SY5Y cells. The total lysates were immunoprecipitated with the anti-IgG or anti-Trx1 antibody and immunoblotted with anti-nitrotyrosine (n = 3). (G) Pull-down analysis of binding between TMP and Trx1 or Trx1^Y49A^ in SH-SY5Y cells (n = 3). EV, empty vehicle. (H) Trx1 activity in Trx1-OE or Trx1^Y49A^-OE SH-SY5Y cells (n = 3). (I) TMP led to the conformational change of Trx1 through the tryptophan fluorescence quenching study. (J) CD spectra analysis for TMP-mediated Trx1 conformational change. (K) Secondary structure composition of Trx1 as analyzed by the BeStSel method. (L) Preferential binding mode between TMP and Trx1 according to docking simulation. The amino acid residues involved in interactions are labeled. (M) The 3D structural conformation of Trx1 was analyzed during the 40 ns molecular dynamics simulation period. Data were presented as mean ± SD. n.s., *P* > 0.05, ^#^*P* < 0.05, ^##^*P* < 0.01 compared with control group; n.s., *P* > 0.05, ∗*P* < 0.05, ∗∗*P* < 0.01 compared with OGD/R group or Trx1-OE group.

Having identified Trx1 as a functional target, we next investigated whether TMP modulates its enzymatic activity. In SH-SY5Y cells subjected to OGD/R, Trx1 activity was significantly suppressed, whereas TMP treatment concentration-dependently restored its activity, with recovery rates of 29.65%, 36.18%, and 66.16% at concentrations of 5, 10, and 20 μM, respectively (Fig. 5A). Given that Trx1 activity can be regulated by PTMs or changes in expression levels [46], we assessed Trx1 protein levels and observed no significant alterations following OGD/R or TMP treatment (Fig. 5B), suggesting a PTM-based regulatory mechanism. Trx1 is known to undergoes multiple PTMs, including C32/C35 oxidation, Y49 nitration [20], C69 *S*-nitrosylation [47], and C73 glutathionylation [48]. To determine whether TMP directly engages Trx1 to modulate these PTMs and thereby restore its activity, we mapped the binding sites of TMP on Trx1. The photo-crosslinked Trx1 complex was subjected to enzymatic digestion and LC-MS/MS analysis. Upon UV irradiation, TMP-P generates a reactive carbene intermediate that inserts into C–H bonds (Fig. 5C), enabling covalent capture and subsequent site-specific identification [49,50]. Seven residues (S44, S46, E47, L97, E98, E103, and L104) were identified based on mass shifts of +138.0681 Da or +272.1525 Da, corresponding to the oct-7-ynoic acid moiety and the intact photoactivated tag, respectively (Fig. 5D, Supplementary Fig. S10). Strikingly, all these residues cluster in close proximity to Y49 (Fig. 5E), a site whose nitration is known to inhibit Trx1 activity [20]. We therefore hypothesized that TMP enhances Trx1 activity by preventing Y49 nitration. Consistent with this, IP assays revealed that OGD/R increased Trx1 nitration, which was effectively suppressed by TMP treatment (Fig. 5F). Moreover, IP coupled with MS analysis was employed to directly verify site-specific nitration of Trx1 under OGD/R conditions. MS spectra confirmed that OGD/R induced a clear mass shift of +44.9851 Da on Y49-containing peptides derived from Trx1, consistent with tyrosine nitration (Supplementary Fig. S11A–B). In contrast, despite comprehensive analysis of the MS data, no modifications were detected at cysteine residues under our experimental conditions (Supplementary Fig. S11C–D), indicating that cysteines do not undergo detectable PTMs in this context. Furthermore, a Y49A point mutation not only weakened the TMP-Trx1 interaction (Fig. 5G) but also abolished the ability of TMP to activate Trx1 (Fig. 5H), underscoring the functional indispensability of Y49. This observation directly links TMP binding to Y49 with its capacity to prevent nitration-dependent inhibition of Trx1 activity.

To further assess the functional consequences of the Y49A mutation downstream of Trx1 activity, we evaluated mitochondrial function and cell apoptosis. TMRM is a widely used probe for mitochondrial membrane potential, and a decrease in its fluorescence intensity indicates mitochondrial depolarization. Using TMRM staining, we found that OGD/R induced mitochondrial depolarization in SH-SY5Y cells overexpressing wild-type Trx1 (Trx1-OE), which was markedly reversed by TMP treatment; however, TMP did not restore the TMRM signal in SH-SY5Y cells overexpressing the Y49A mutant (Trx1^Y49A^-OE) to a comparable extent (Supplementary Fig. S12A). Hoechst 33258 staining further revealed that TMP significantly reduced OGD/R-induced apoptosis in Trx1-OE cells but showed no significant effect in Trx1^Y49A^-OE cells (Supplementary Fig. S12B). These data suggest that the Y49A mutation impairs TMP-mediated protection of mitochondrial function and anti-apoptotic effects.

Given that Y49 is distant from the catalytic site, we hypothesized that TMP might regulate Trx1 activity through an allosteric mechanism. To test this, we first employed intrinsic tryptophan fluorescence spectroscopy to probe conformational changes in Trx1. A decrease in fluorescence intensity was observed upon TMP treatment, suggesting that TMP induced a conformational change in Trx1 (Fig. 5I). We further characterized secondary structural changes using CD spectroscopy. Under native conditions, Trx1 contained 31.3% regular α-helix, 11.6% distorted α-helix, and 30.0% β-sheet. Titration of TMP into Trx1 led to a progressive decrease in the molar ellipticity at 192.8 and 200.8 nm, reflecting a concentration-dependent reduction in overall helicity. When the molar ratio of Trx1 to TMP reached 1:200, the secondary structure of Trx1 shifted to 20.1% regular α-helix, 21.9% distorted α-helix, and 20.6% β-sheet (Fig. 5J and K), confirming that TMP induced conformational rearrangements in Trx1. To gain structural insight into this allosteric change, we performed molecular docking and all-atom MD simulations. Docking positioned TMP stably adjacent to Y49, forming a π-π stacking interaction with the aromatic ring of Y49 and a hydrogen bond with K48 (Fig. 5L). Subsequent MD simulations revealed that TMP binding promotes a conformational shift that significantly increases the spatial separation between the catalytic cysteines C32 and C35, a structural change that likely stabilizes the reduced, active form of Trx1 (Fig. 5M). In summary, these data demonstrate that TMP binds to Y49 of Trx1, inhibits its nitration, and allosterically activates Trx1.

### 3.6 TMP stabilizes the Trx1-ASK1 complex and suppresses redox-dependent signaling

**Fig. 6:**
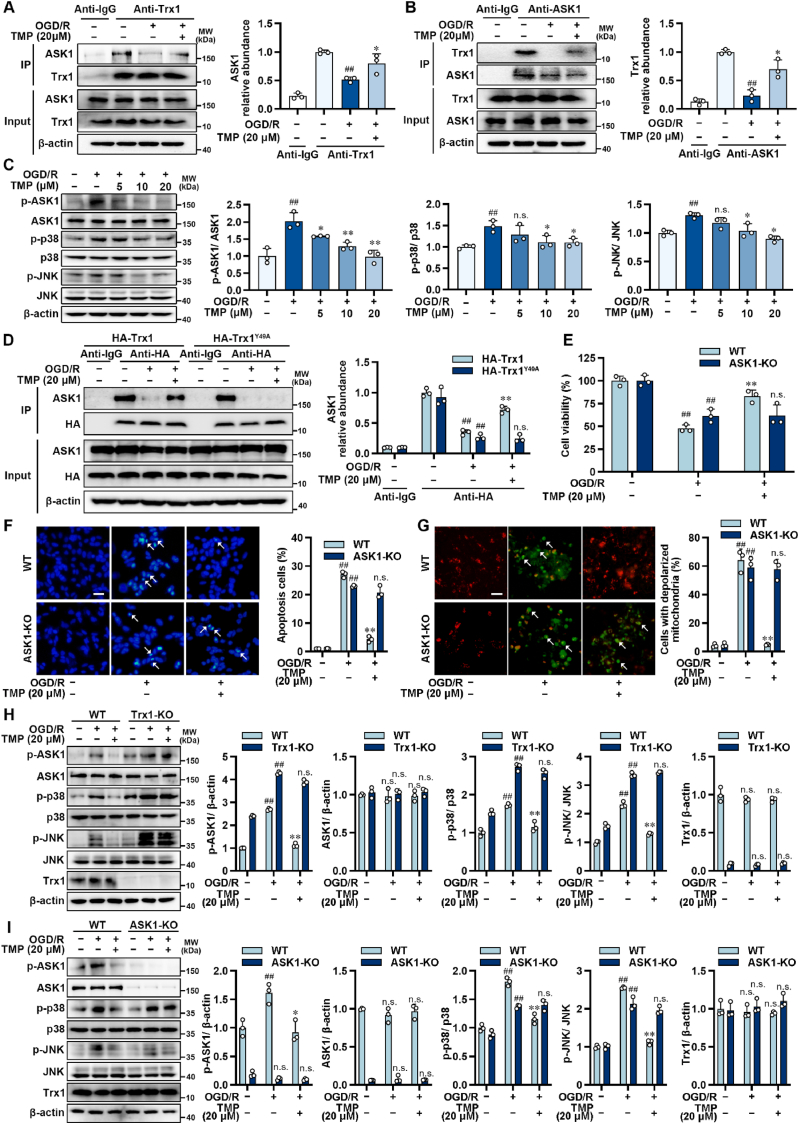
TMP stabilizes the Trx1-ASK1 complex and suppresses redox-dependent signaling. (A) Binding of Trx1 and ASK1 determined by co-IP assays in SH-SY5Y cells. The total lysates were immunoprecipitated with the anti-Trx1 antibody and immunoblotted with anti-ASK1 (n = 3). (B) Binding of ASK1 and Trx1 determined by co-IP assays in SH-SY5Y cells. The total lysates were immunoprecipitated with the anti-ASK1 antibody and immunoblotted with anti-Trx1 (n = 3). (C) Western blotting of p-ASK1, ASK1, p-p38, p38, *p*-JNK, JNK levels and their quantitation in SH-SY5Y cells (n = 3). (D) Binding of Trx1 or Trx1^Y49A^ and ASK1 determined by co-IP assays in SH-SY5Y cells. The total lysates were immunoprecipitated with the anti-HA antibody and immunoblotted with anti-ASK1 (n = 3). (E) Cell viability of WT and ASK1-KO SH-SY5Y cells determined by CCK-8 assay (n = 3). (F) Hoechst 33258 staining of WT and ASK1-KO SH-SY5Y cells (scale bar: 50 μm, n = 3). (G) JC-1 staining of WT and ASK1-KO SH-SY5Y cells (scale bar: 50 μm, n = 3). (H) Western blotting of p-ASK1, ASK1, p-p38, p38, *p*-JNK, JNK, Trx1 levels and their quantification in WT and Trx1-KO SH-SY5Y cells (n = 3). (I) Western blotting of p-ASK1, ASK1, p-p38, p38, *p*-JNK, JNK, Trx1 levels and their quantification in WT and ASK1-KO SH-SY5Y cells (n = 3). Data were presented as mean ± SD. n.s., *P* > 0.05, ^##^*P* < 0.01 compared with control group; n.s., *P* > 0.05, ∗*P* < 0.05, ∗∗*P* < 0.01 compared with OGD/R group.

We next explored the functional consequences of TMP-induced Trx1 activation. As Trx1 binds to and inhibits ASK1 via its redox-active cysteines (C32/C35) [51], we hypothesized that the active Trx1 conformation stabilized by TMP might promote Trx1-ASK1 complex formation. Co-IP assays confirmed that Trx1 and ASK1 form a constitutive complex under basal conditions, which was significantly disrupted by OGD/R. Importantly, TMP treatment restored Trx1-ASK1 binding (Fig. 6A and B). Consistent with the model that Trx1-ASK1 dissociation activates the ASK1-p38/JNK signaling axis, OGD/R induced phosphorylation of ASK1, p38, and JNK, all of which were suppressed by TMP (Fig. 6C). The critical dependence of this stabilization on TMP binding to Y49 was demonstrated by the loss of Trx1-ASK1 complex formation in cells expressing the Y49A Trx1 mutant (Fig. 6D). Genetic ablation of *ASK1* abolished the neuroprotective effects of TMP (Fig. 6E–G), and loss of either *Trx1* or *ASK1* prevented TMP-mediated suppression of p-ASK1, p-p38, and *p*-JNK (Fig. 6H and I). These results indicate that TMP confers neuroprotection by enhancing Trx1-ASK1 binding and inhibiting downstream redox-sensitive signaling.

### 3.7 TMP alleviates ischemic injury by suppressing Trx1 nitration and restoring Trx1 activity

**Fig. 7:**
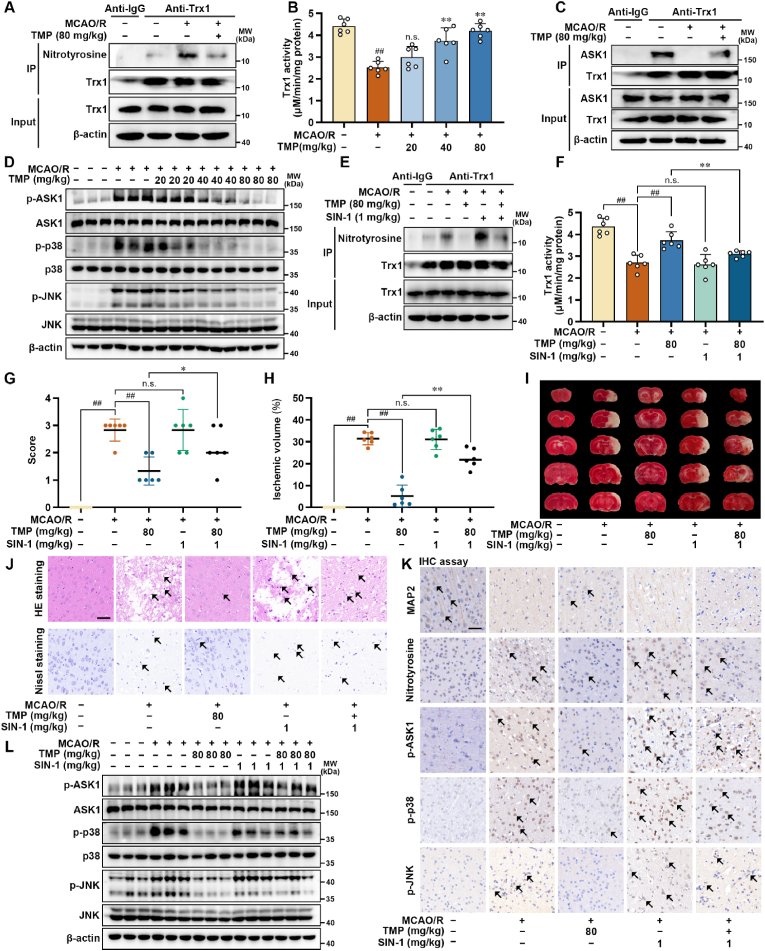
TMP alleviates ischemic injury by suppressing Trx1 nitration and preserving redox homeostasis. (A) Trx1 nitration determined by IP assays in rat brains. The total lysates were immunoprecipitated with the anti-Trx1 antibody and immunoblotted with anti-nitrotyrosine. (B) Trx1 activity in the tissue of the peri-infract area of the rat brains (n = 6). Data were presented as mean ± SD. ^##^*P* < 0.01 compared with sham group; n.s., *P* > 0.05, ∗∗*P* < 0.01 compared with MCAO/R group. (C) Binding of Trx1 and ASK1 determined by co-IP assays in rat brains. The total lysates were immunoprecipitated with the anti-Trx1 antibody and immunoblotted with anti-ASK1. (D) Western blotting of p-ASK1, ASK1, p-p38, p38, *p*-JNK, JNK levels in the brain of rats. (E) Trx1 nitration determined by IP assays in the rat brains. (F) Trx1 activity in the tissue of the peri-infract area of rat brains (n = 6). (G) Neurological deficient scores at day 3 (n = 6). (H) SIN-1 blocks the protective effect of TMP on MCAO/R-induced ischemic volume (n = 6). Data were presented as mean ± SD. n.s., *P* > 0.05, ^##^*P* < 0.01 compared with MCAO/R group; ∗∗*P* < 0.01 compared with TMP group. (I) Representative photographs of TTC stained coronal sections. (J) HE and Nissl staining showing morphological neuronal changes. Arrows indicate damaged neurons (scale bar: 50 μm). (K) MAP2, nitrotyrosine, p-ASK1, p-p38, and *p*-JNK contents determined by IHC assays (scale bar: 50 μm). Arrows indicate positive cells. (L) Western blotting of p-ASK1, ASK1, p-p38, p38, *p*-JNK, and JNK levels in rat brains.

To validate the pathophysiological relevance of Trx1 nitration in vivo, we assessed its nitration status and enzymatic activity in MCAO/R rat brains. IP analysis revealed that MCAO/R significantly increased Trx1 nitration, an effect that was attenuated by TMP treatment (Fig. 7A, Supplementary Fig. S13A). Consistently, Trx1 activity was markedly suppressed following MCAO/R and restored by TMP in a dose-dependent manner, with recovery rates of 25.04%, 65.04%, and 88.67% at doses of 20, 40, and 80 mg/kg, respectively (Fig. 7B). Importantly, TMP enhanced the association between Trx1 and ASK1 (Fig. 7C) and significantly suppressed activation of the ASK1-p38/JNK signaling cascade (Fig. 7D, Supplementary Fig. S13B).

To further substantiate the causal role of Trx1 nitration, we employed SIN-1, a peroxynitrite donor known to induce Trx1 nitration and inactivation [20,52]. LC-MS/MS analysis confirmed that SIN-1 treatment did not induce detectable modifications at cysteine residues of Trx1 under our experimental conditions (Supplementary Fig. S14A–B). SIN-1 administration effectively abrogated TMP-mediated suppression of Trx1 nitration (Fig. 7E, Supplementary Fig. S13C) and markedly impaired the ability of TMP to restore Trx1 activity (Fig. 7F). We next evaluated whether SIN-1 could counteract the neuroprotective effects of TMP in vivo. Neurological function assessments revealed severe deficits following MCAO/R, which were significantly ameliorated by TMP treatment; however, this improvement was substantially diminished upon co-administration of SIN-1 (Fig. 7G). Consistent with these observations, SIN-1 attenuated the ability of TMP to reduce cerebral infarct volume (Fig. 7H and I). Histopathological evaluations using HE and Nissl staining indicated that TMP-mediated preservation of ischemic cortical neurons was negated by SIN-1 co-treatment (Fig. 7J, Supplementary Fig. S13D). Immunohistochemical analysis of the dendritic marker MAP2 revealed that TMP enhanced neuronal structural integrity, an effect that was diminished by SIN-1 (Fig. 7K, Supplementary Fig. S13E). Quantitative analysis of 3-nitrotyrosine immunoreactivity showed that TMP mitigated the elevated nitrative stress induced by MCAO/R, whereas SIN-1 co-treatment blunted this effect (Fig. 7K, Supplementary Fig. S13E). Western blotting and immunohistochemical analyses further demonstrated that TMP suppressed the MCAO/R-induced phosphorylation of ASK1, p38, and JNK, and that SIN-1 interfered with TMP-mediated inhibition of the ASK1-p38/JNK pathway (Fig. 7K and L, Supplementary Fig. S13E and F). In summary, these comprehensive in vivo results unequivocally demonstrate that TMP alleviates ischemic injury by inhibiting Trx1 nitration, preserving its redox activity, and thereby suppressing downstream redox-sensitive signaling.

## 4 Discussion

Natural products represent a rich reservoir of therapeutic agents [53,54], yet their clinical translation is often hindered by an incomplete understanding of their molecular targets and mechanisms of action. In this study, we applied a chemical proteomics strategy that integrates a bifunctional photoaffinity probe with SILAC-ABPP to systematically identify Trx1 as a direct functional target of TMP. This approach enables the capture of transient, non-covalent drug-target interactions and facilitates precise mapping of target engagement within native cellular environments. Through competitive binding coupled with SILAC-based quantification, we reliably distinguished specific targets from nonspecific binders, underscoring the robustness of this strategy for interrogating the interactome of non-covalent bioactive compounds.

The long-standing clinical use of TMP for ischemic stroke has been constrained by the absence of a defined molecular target, impeding both mechanistic understanding and rational drug optimization [22,55,56]. Our identification of Trx1 as a principal target of TMP addresses this critical knowledge gap. This finding not only provides a mechanistic foundation for TMP's established efficacy but also opens new avenues for developing TMP derivatives with improved target affinity and pharmacokinetic properties. Moreover, given the central role of Trx1 in a spectrum of redox-related pathologies—including neurodegenerative [8,9], cardiometabolic [[57], [58], [59]], and diabetic conditions [60]—its identification as a TMP target offers a plausible molecular basis for the broad pharmacological activities attributed to this compound, a hypothesis that warrants further investigation.

Trx1 is the direct and specific target through which TMP exerts its neuroprotective effects against I/R injury. This conclusion is supported by a convergent, multi-layered experimental strategy. Although TMP is a pleiotropic compound that may engage distinct targets in other pathological contexts, our data establish Trx1 as its primary and necessary target within the specific framework of I/R injury. While our focused off-target validation (including TrxR1, Keap1, and VDAC1) strengthens confidence in this conclusion, it is inherently limited in scope and does not exhaustively exclude all possible interactions. By specifically activating Trx1, TMP enhances the cellular capacity to counteract I/R-induced oxidative stress, thereby attenuating downstream redox-sensitive signaling. This mechanism distinguishes TMP from non-specific antioxidants and aligns with the therapeutic strategy of selectively bolstering endogenous cytoprotective systems.

A pivotal finding of this work is the identification of Y49 on Trx1 as a druggable allosteric site and the characterization of TMP as its first-in-class allosteric activator. Conventional strategies targeting the redox-active C32–C35 motif are often compromised by oxidative inactivation and potential off-target effects [61]. In contrast, TMP engagement at Y49 enables effective modulation of Trx1 activity without direct interference at the catalytic site. This allosteric mechanism offers enhanced selectivity and functional resilience under nitrative stress conditions, establishing a novel paradigm for therapeutic targeting of Trx1.

Mechanistically, we demonstrate that TMP binding to Y49 not only antagonizes its nitrative modification but also induces a defined conformational shift in Trx1, as evidenced by spectroscopic and computational analyses. We propose that this structural rearrangement increases the spatial separation between C32 and C35, thereby stabilizing the reduced, active form of Trx1. This conformational activation has direct functional consequences. Because the interaction between Trx1 and ASK1 is redox-dependent and specifically requires the reduced state of C32/C35 [62], TMP-induced stabilization of the active Trx1 conformation promotes Trx1-ASK1 complex formation. This, in turn, suppresses ASK1-mediated activation of the downstream kinases p38 and JNK, ultimately attenuating the apoptotic signaling cascade and mitigating neuronal injury. Thus, Y49 emerges as a therapeutically targetable site that integrates nitrative defense with allosteric control of Trx1 function. Our work not only elucidates a precise molecular mechanism by which TMP protects against cerebral I/R injury but also highlights the broader relevance of targeting this regulatory nexus in ischemic stroke and other disorders driven by nitrative and oxidative stress. Notably, the functional characterization of Y49 in this study relies primarily on cellular and pharmacological intervention strategies. The lack of Y49A knock-in animals means that the in vivo consequences of this mutation for neurological recovery following ischemic stroke remain to be directly validated. The generation of such models will be essential for a comprehensive assessment of Y49 as a therapeutic target.

Nitrated proteins are increasingly recognized as active contributors to pathology, rather than mere biomarkers of oxidative stress [7,63,64]. Our data demonstrate that nitrative stress is a key driver of neuronal damage in cerebral ischemia, and that TMP significantly attenuates 3-nitrotyrosine formation in the ischemic cortex while preserving Trx1 activity, underscoring the central role of its anti-nitrative activity in conferring neuroprotection. The finding that SIN-1 co-treatment prevented TMP-mediated neuroprotection in vivo further supports the functional relevance of this anti-nitrative mechanism. We acknowledge the complexity of peroxynitrite chemistry, which under certain conditions can also target cysteine residues. However, within the scope of our study, the convergent evidence supports the conclusion that Trx1 inactivation under our experimental conditions is predominantly mediated by nitration at Y49. While other PTMs such as *S*-nitrosylation and *S*-glutathionylation may influence Trx1 function under different redox conditions, the present study specifically focuses on nitration as the key pathological event and therapeutic target in ischemic stroke. The potential interplay between nitration and these other modifications represents an important area for future investigation. To our knowledge, this study is the first to report a small molecule that specifically counteracts Trx1 nitration and preserves its reductase function in cerebral ischemia, presenting a novel pharmacological strategy to combat nitrative damage in stroke.

## 5 Conclusion

In summary, this study identifies Trx1 as the direct functional target responsible for TMP's neuroprotective effects. We delineate a novel mechanism whereby TMP binds to Y49 on Trx1, inhibits its nitrative inactivation, and functions as an allosteric activator. This action stabilizes the Trx1-ASK1 complex and suppresses the downstream ASK1-p38/JNK signaling pathway. Genetic or pharmacological disruption of this axis completely abolishes TMP's protective efficacy. Our findings not only provide a long-sought mechanistic explanation for TMP's clinical utility but also unveil Y49 as a druggable allosteric site on Trx1, offering a new redox-targeted therapeutic strategy for ischemic stroke and related disorders characterized by nitrative stress.

## CRediT authorship contribution statement

**Meizhu Guo:** Conceptualization, Data curation, Formal analysis, Investigation, Visualization, Writing – original draft. **Huan Guo:** Validation. **Wenshuo Ren:** Validation. **Xiaonian Wang:** Investigation. **Xing Wang:** Supervision. **Yang Liu:** Writing – review & editing. **Weijun Kong:** Resources, Supervision. **Qiang Guo:** Conceptualization, Funding acquisition, Methodology, Project administration, Resources, Supervision, Writing – review & editing.

## Declaration of competing interest

The authors declare that they have no known competing financial interests or personal relationships that could have appeared to influence the work reported in this paper.

## Data Availability

Data will be made available on request.
