# Supplementary material for: Allosteric activation of Trx1 by antagonizing nitrative modification at tyrosine 49 confers neuroprotection against ischemic stroke
Source: Redox Biol. 2026 Jun 4;95:104246. doi: 10.1016/j.redox.2026.104246 (PMC13276151; doi:10.1016/j.redox.2026.104246)
Supplement: Multimedia component 1 [file mmc1.docx]

Supplementary material

**Allosteric activation of Trx1 by antagonizing nitrative modification at tyrosine 49 confers neuroprotection against ischemic stroke**

**CONTENT**

**Fig. S1.** TMP reduced OGD/R-induced ROS production in SH-SY5Y cells, as determined by DCFH-DA staining assay.

**Fig. S2.** Synthesis of TMP-P.

**Fig. S3.** HR-ESI-MS spectrum of TMP-P.

**Fig. S4.** ^1^H-NMR (CDCl_3_, 500 MHz) spectrum of TMP-P.

**Fig. S5.** ^13^C-NMR (CDCl_3_, 125 MHz) spectrum of TMP-P.

**Fig. S6.** ^1^H–^1^H COSY spectrum of TMP-P.

**Fig. S7.** HSQC spectrum of TMP-P.

**Fig. S8.** HMBC spectrum of TMP-P.

**Fig. S9.** HPLC-UV (280 nm) chromatogram of TMP-P.

**Fig. S10.** LC-MS/MS analysis of the TMP-P-bound fragment derived from Trx1.

**Fig. S11.** LC‑MS/MS identification of Trx1-derived peptides.

**Fig. S12.** Y49A mutation impairs TMP-mediated protection against OGD/R-induced mitochondrial dysfunction and apoptosis.

**Fig. S13.** LC-MS/MS analysis of Trx1 cysteine‑containing peptides following SIN‑1 treatment.

**Fig. S14.** TMP exerts anti-ischemic stroke effect in vivo by targeting Trx1.

**Fig. S15.** Uncropped western blot membranes for Figure 2E to Figure 7L.

**Table S1.** High-confidence protein candidates identified from in situ photocrosslinking experiments.

**Table S2.** High-confidence protein candidates identified from in vitro photocrosslinking experiments.


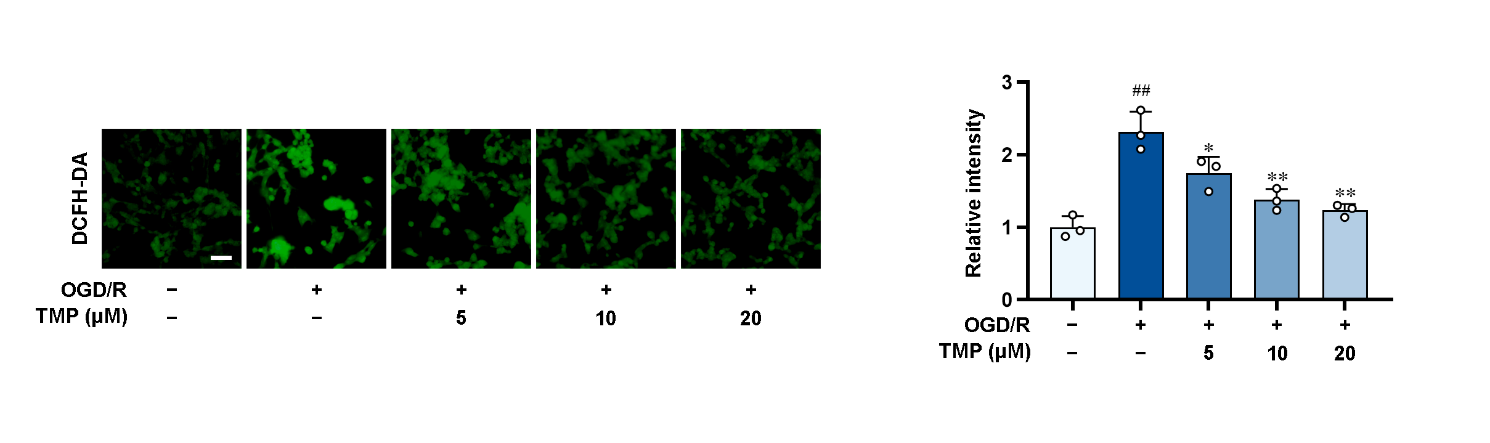


**Fig. S1**. TMP reduced OGD/R-induced ROS production in SH-SY5Y cells, as determined by DCFH-DA staining assay (scale bar: 50 μm, n = 3). Data were presented as mean ± SD. ^##^*P* < 0.01 compared with control group; ^*^*P* < 0.05, ^**^*P* < 0.01 compared with OGD/R group.

# Fig. S2. Synthesis of TMP-P.

# Fig. S3. HR-ESI-MS spectrum of TMP-P.

# Fig. S4. ^1^H NMR (CDCl_3_, 500 MHz) spectrum of TMP-P.

# Fig. S5. ^13^C NMR (CDCl_3_, 125 MHz) spectrum of TMP-P.

# Fig. S6. ^1^H–**^1^**H COSY spectrum of TMP-P.

# Fig. S7. HSQC spectrum of TMP-P.

# Fig. S8. HMBC spectrum of TMP-P.

# Fig. S9. HPLC-UV (280 nm) chromatogram of TMP-P.


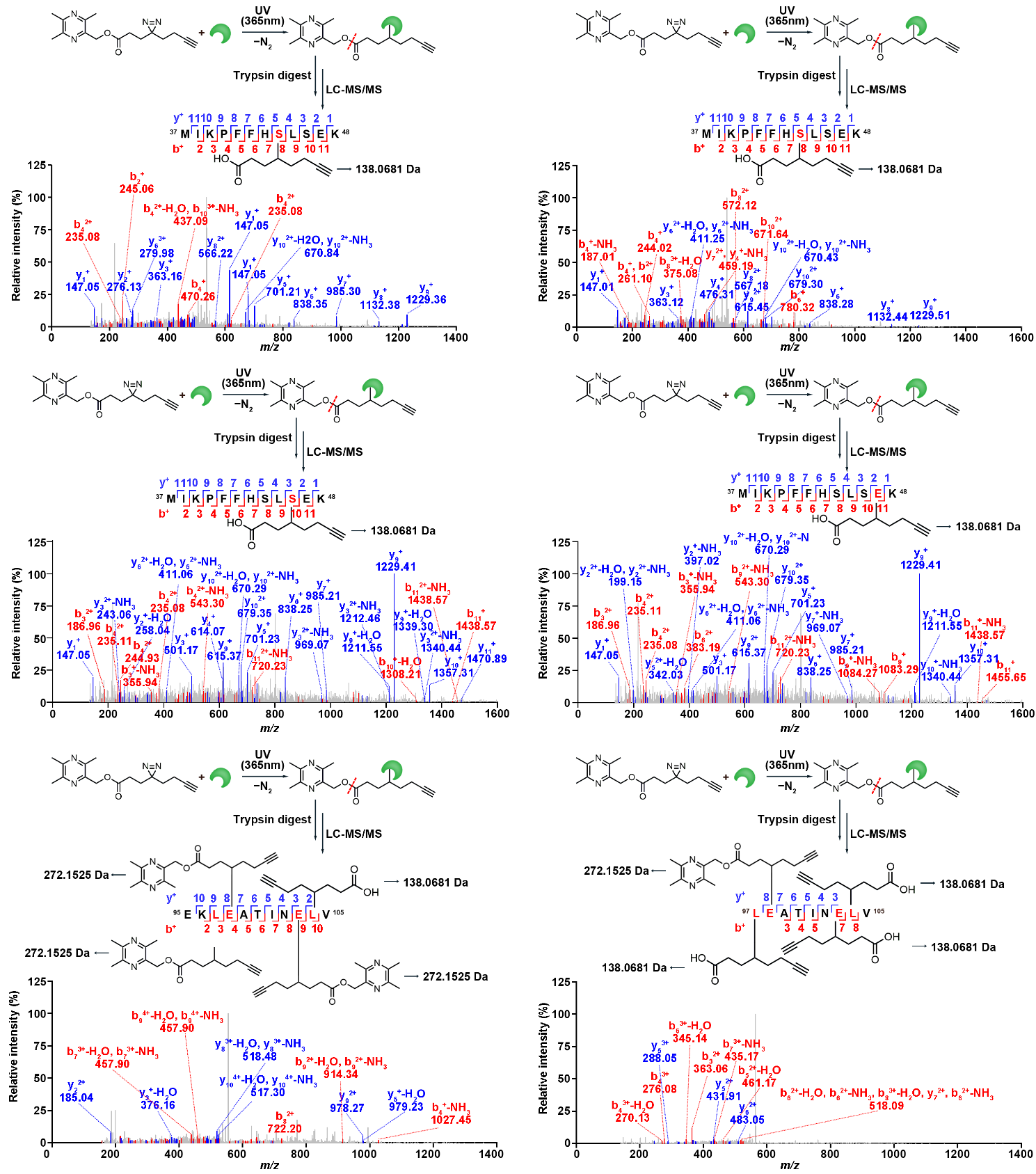


# Fig. S10. LC-MS/MS analysis of the TMP-P-bound peptides derived from Trx1.


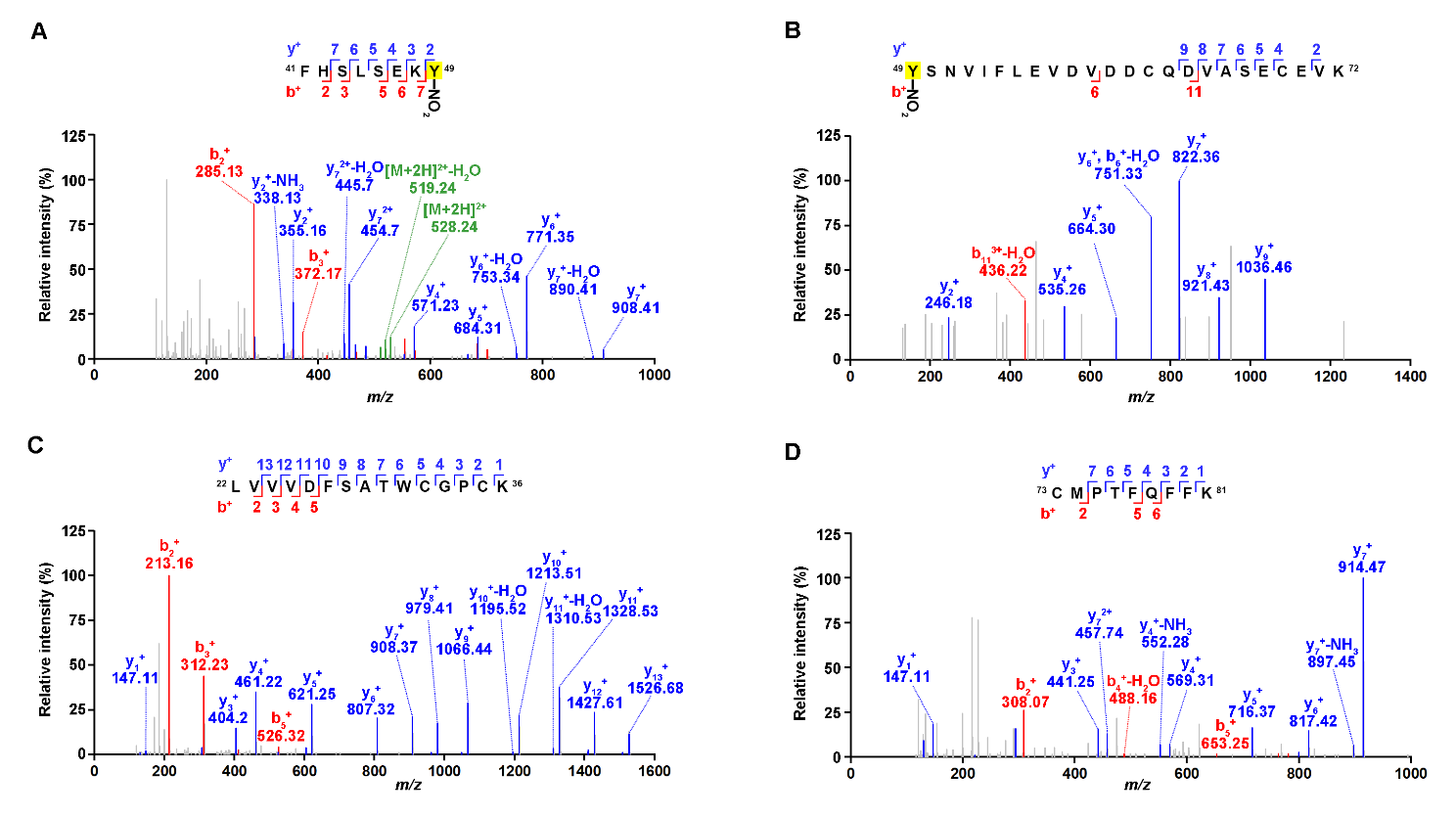


# Fig. S11. LC‑MS/MS identification of Trx1-derived peptides. (A) LC‑MS/MS identification of the chymotryptic peptide derived from Trx1 following IP, showing nitration at Y49. (B) LC‑MS/MS identification of tryptic peptide derived from Trx1 following IP, showing a mass shift consistent with nitration. (C, D) LC‑MS/MS identification of peptides derived from Trx1 following IP, showing no detectable modification signals at cysteine residues.


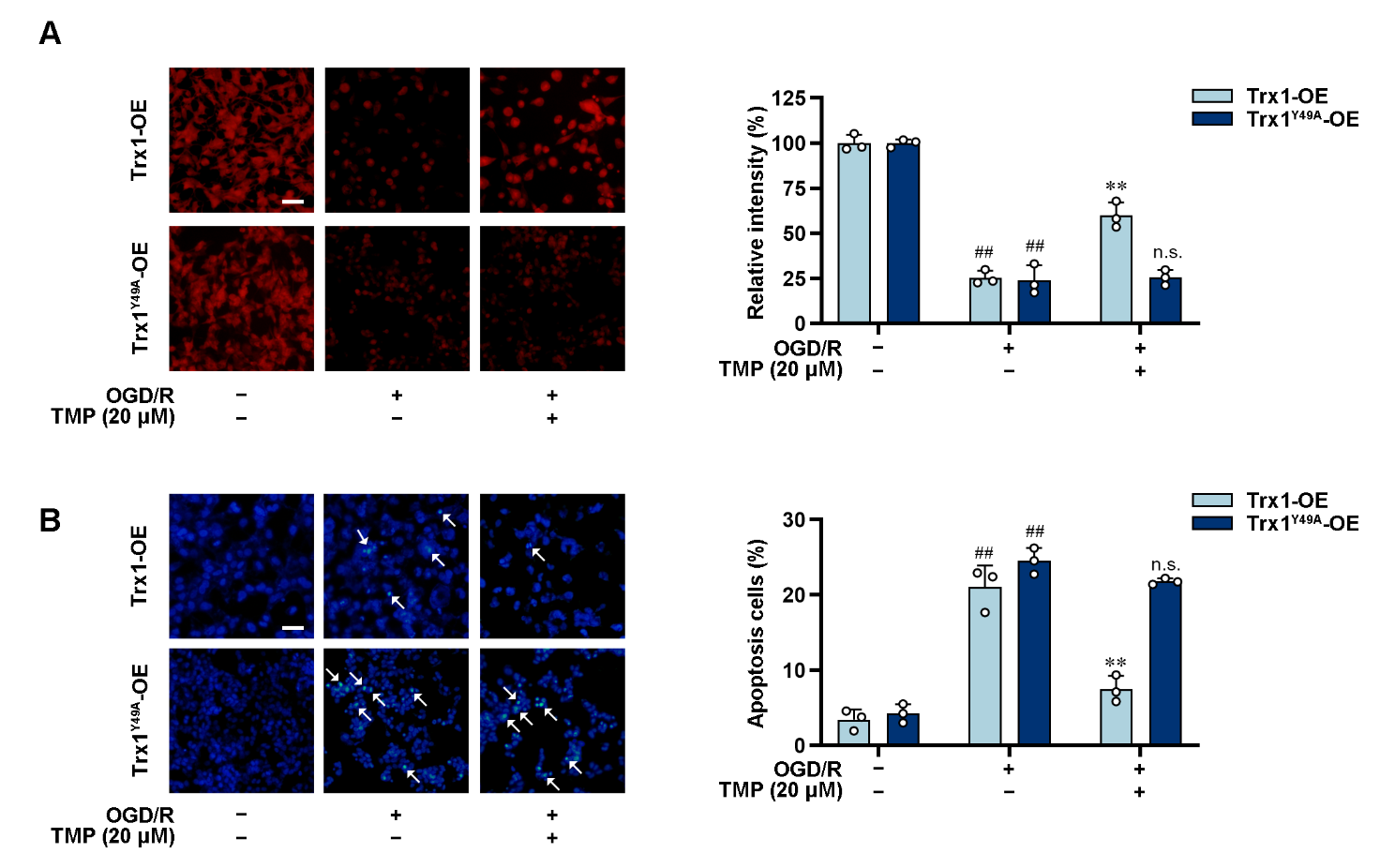


**Fig. S12.** Y49A mutation impairs TMP-mediated protection against OGD/R-induced mitochondrial dysfunction and apoptosis (scale bar: 50 μm, n = 3). (A) TMRM staining for mitochondrial membrane potential. (B) Hoechst 33258 staining for nuclear apoptosis (scale bar: 50 μm, n = 3). Arrows indicate brightly stained condensed nuclei for apoptosis. Data were presented as mean ± SD. ^##^*P* < 0.01 compared with control group; n.s., *P* > 0.05, ^**^*P* < 0.01 compared with OGD/R group.

**
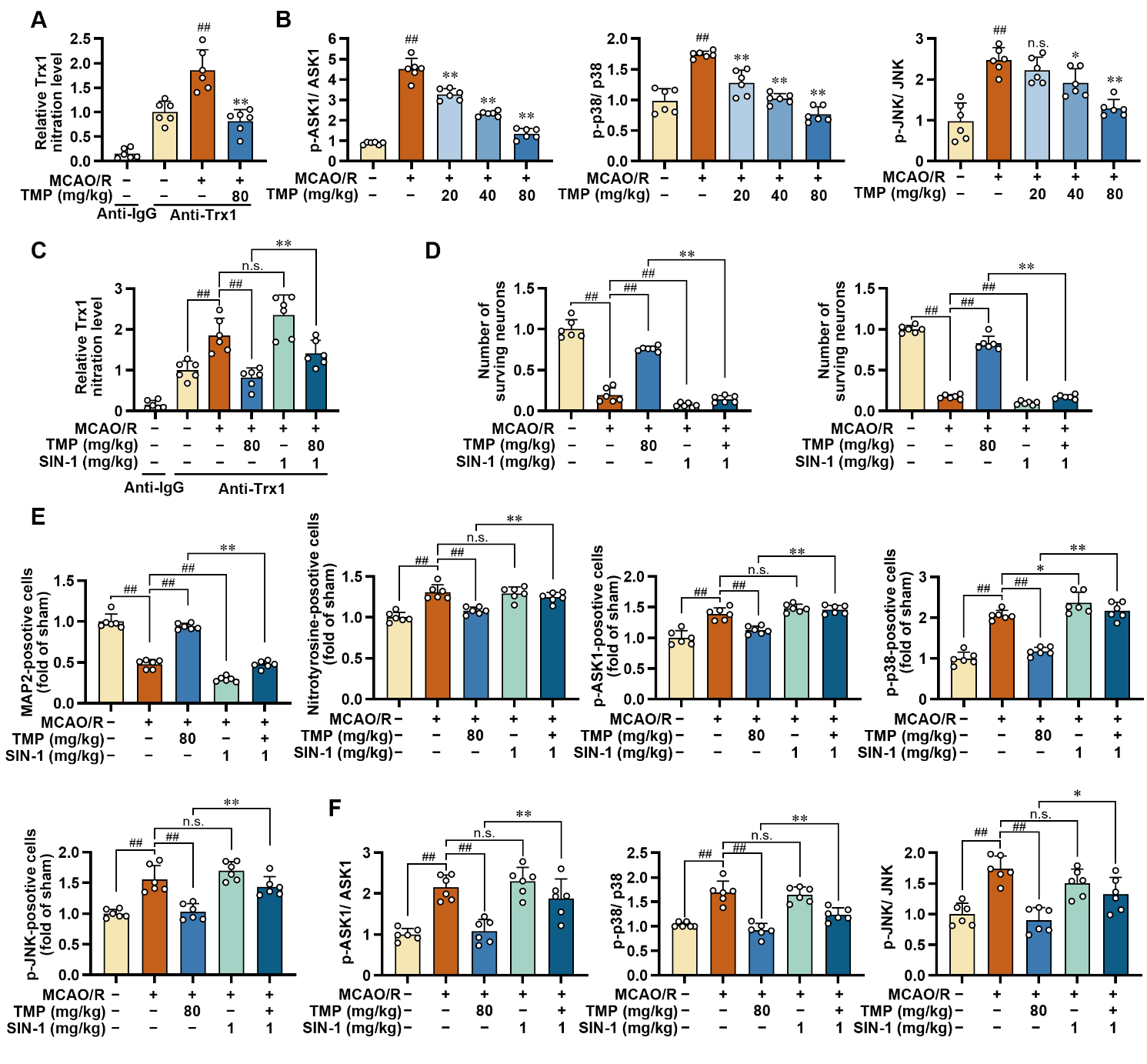
**

# Fig. S13. TMP exerts anti-ischemic stroke effect in vivo by targeting Trx1. (A) Quantification of Trx1 nitration in rat brains, as verified by IP assay and determined by western blot (n = 6). (B) Quantitation of western blot analysis of p-ASK1, ASK1, p-p38, p38, p-JNK, and JNK expression in rat brains (n = 6). Data were presented as mean ± SD. ^##^*P* < 0.01 compared with sham group; n.s., *P* > 0.05, ^**^*P* < 0.01, ^*^*P* < 0.05 compared with MCAO/R group. (C) Quantification of Trx1 nitration in rat brains, as verified by IP assay and determined by western blot (n = 6). (D) Quantitation of HE and Nissl staining showing morphological neuronal changes (n = 6). (E) Quantitation of IHC assay for MAP2, nitrotyrosine, p-ASK1, p-p38, and p-JNK expression (n = 6). (F) Quantitation of western blot analysis of p-ASK1, ASK1, p-p38, p38, p-JNK, and JNK expression in rat brains (n = 6). Data were presented as mean ± SD. n.s., *P* > 0.05, ^##^*P* < 0.01 compared with MCAO/R group; ^*^*P* < 0.05, ^**^*P* < 0.01 compared with TMP group.


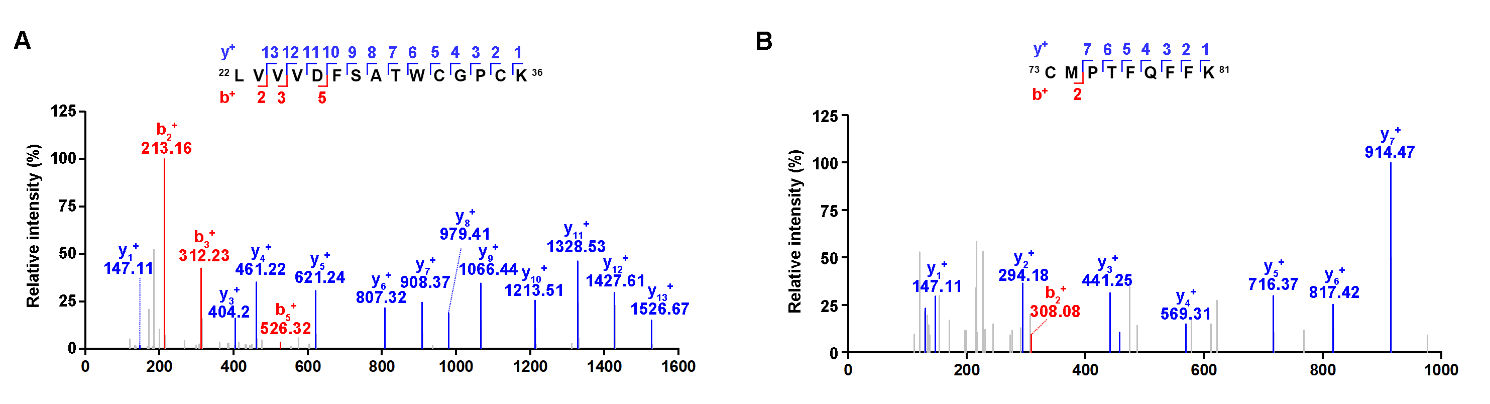


# Fig. S14. LC-MS/MS analysis of Trx1 cysteine‑containing peptides following SIN‑1 treatment, showing no detectable modifications at cysteine residues.


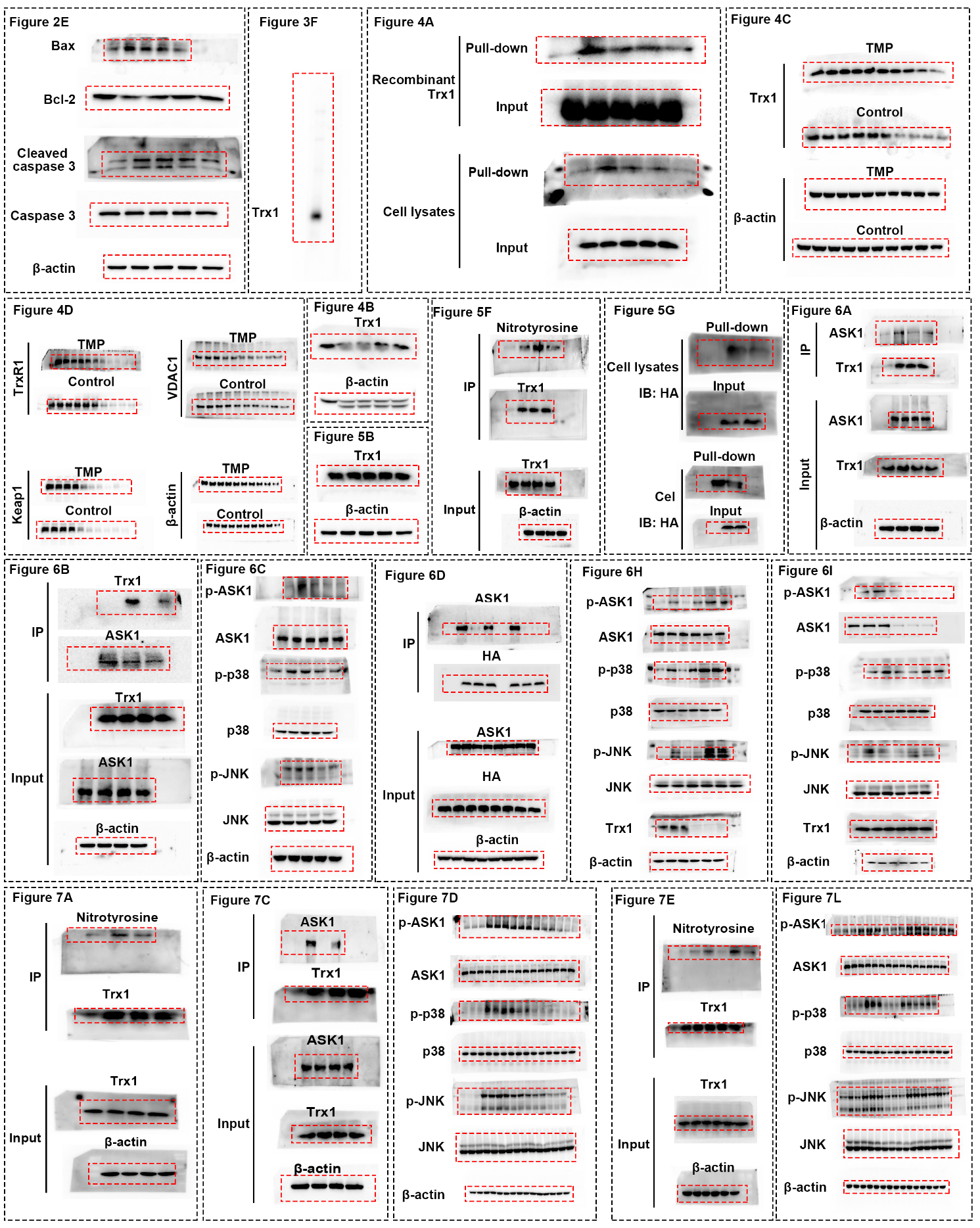


# Fig. S15. Uncropped western blot membranes for Figure 2E to Figure 7L.

# Table S1. High-confidence protein candidates identified from in situ photocrosslinking experiments.

|  | ID | Protein | Abundance Ratio (log2): (Light) / (Heavy) | *P*-Value (−log10): (Light) / (Heavy) | Coverage (%) | Peptides | Peptide spectrum matches |
| --- | --- | --- | --- | --- | --- | --- | --- |
| 1 | Q8WXA9 | SREK1 | 6.64 | 15.89 | 2 | 1 | 1 |
| 2 | Q8TB61 | SLC35B2 | 6.64 | 15.89 | 3 | 1 | 1 |
| 3 | Q12789 | GTF3C1 | 6.64 | 15.89 | 1 | 1 | 1 |
| 4 | P31350 | RRM2 | 6.64 | 15.89 | 2 | 1 | 1 |
| 5 | O95801 | TTC4 | 6.64 | 15.89 | 3 | 1 | 1 |
| 6 | Q92797 | SYMPK | 6.64 | 15.89 | 1 | 1 | 1 |
| 7 | Q5JVF3 | PCID2 | 6.64 | 15.89 | 3 | 1 | 1 |
| 8 | P40937 | RFC5 | 6.64 | 15.89 | 2 | 1 | 1 |
| 9 | Q8WVB6 | CHTF18 | 6.64 | 15.89 | 1 | 1 | 1 |
| 10 | Q5W5X9 | TTC23 | 6.64 | 15.89 | 5 | 1 | 1 |
| 11 | Q96EV2 | RBM33 | 6.64 | 15.89 | 1 | 1 | 2 |
| 12 | Q9H4A4 | RNPEP | 6.64 | 15.89 | 2 | 1 | 1 |
| 13 | P43378 | PTPN9 | 6.64 | 15.89 | 2 | 1 | 1 |
| 14 | P42574 | CASP3 | 6.64 | 15.89 | 4 | 1 | 1 |
| 15 | Q9H269 | VPS16 | 6.64 | 15.89 | 1 | 1 | 1 |
| 16 | Q92522 | H1FX | 6.64 | 15.89 | 7 | 1 | 1 |
| 17 | Q9H270 | VPS11 | 6.64 | 15.89 | 1 | 1 | 1 |
| 18 | Q9H9B1 | EHMT1 | 6.64 | 15.89 | 1 | 1 | 1 |
| 19 | Q05639 | EEF1A2 | 6.64 | 15.89 | 11 | 6 | 86 |
| 20 | P35658 | NUP214 | 6.64 | 15.89 | 0 | 1 | 1 |
| 21 | Q9H0A0 | NAT10 | 6.64 | 15.89 | 1 | 1 | 2 |
| 22 | P43487 | RANBP1 | 6.64 | 15.89 | 4 | 1 | 1 |
| 23 | Q12888 | TP53BP1 | 6.64 | 15.89 | 0 | 1 | 2 |
| 24 | Q06587 | RING1 | 6.64 | 15.89 | 3 | 1 | 1 |
| 25 | P35237 | SERPINB6 | 6.64 | 15.89 | 4 | 1 | 1 |
| 26 | P49902 | NT5C2 | 6.64 | 15.89 | 2 | 1 | 1 |
| 27 | Q8NC51 | SERBP1 | 6.64 | 15.89 | 5 | 2 | 2 |
| 28 | Q96GN5 | CDCA7L | 6.64 | 15.89 | 3 | 1 | 1 |
| 29 | P51659 | HSD17B4 | 6.64 | 15.89 | 2 | 1 | 1 |
| 30 | Q96JB2 | COG3 | 6.64 | 15.89 | 2 | 1 | 1 |
| 31 | P48730 | CSNK1D | 6.64 | 15.89 | 2 | 1 | 1 |
| 32 | Q6GYQ0 | RALGAPA1 | 6.64 | 15.89 | 0 | 1 | 1 |
| 33 | Q7Z460 | CLASP1 | 6.64 | 15.89 | 1 | 1 | 1 |
| 34 | Q13601 | KRR1 | 6.64 | 15.89 | 2 | 1 | 1 |
| 35 | P38935 | IGHMBP2 | 6.64 | 15.89 | 1 | 1 | 1 |
| 36 | Q9UJX4 | ANAPC5 | 6.64 | 15.89 | 1 | 1 | 1 |
| 37 | Q9H7E9 | C8orf33 | 6.64 | 15.89 | 7 | 1 | 1 |
| 38 | Q96T60 | PNKP | 4.11 | 15.89 | 2 | 1 | 1 |
| 39 | Q9BRJ2 | MRPL45 | 4.11 | 15.89 | 3 | 1 | 1 |
| 40 | Q9Y6E2 | BZW2 | 2.88 | 15.89 | 2 | 1 | 2 |
| 41 | O15075 | DCLK1 | 2.86 | 15.89 | 1 | 1 | 1 |
| 42 | P31153 | MAT2A | 2.84 | 15.89 | 4 | 1 | 2 |
| 43 | Q9NZJ4 | SACS | 2.83 | 15.89 | 0 | 1 | 1 |
| 44 | P62318 | SNRPD3 | 2.34 | 15.89 | 8 | 1 | 1 |
| 45 | P61106 | RAB14 | 2.25 | 15.89 | 9 | 2 | 8 |
| 46 | Q01469 | FABP5 | 2.15 | 15.89 | 7 | 1 | 1 |
| 47 | Q9BVM2 | DPCD | 1.99 | 15.89 | 4 | 1 | 1 |
| 48 | P62191 | PSMC1 | 1.93 | 15.89 | 2 | 1 | 1 |
| 49 | O15143 | ARPC1B | 1.88 | 15.89 | 6 | 2 | 2 |
| 50 | P29144 | TPP2 | 1.82 | 15.89 | 1 | 2 | 3 |
| 51 | P30086 | PEBP1 | 1.65 | 15.89 | 4 | 1 | 1 |
| 52 | P09884 | POLA1 | 1.77 | 13.96 | 0 | 1 | 1 |
| 53 | Q16836 | HADH | 2.94 | 13.86 | 3 | 1 | 1 |
| 54 | O94888 | UBXN7 | 1.40 | 13.20 | 3 | 1 | 2 |
| 55 | Q9C0J8 | WDR33 | 2.27 | 11.05 | 1 | 1 | 1 |
| 56 | P52597 | HNRNPF | 1.19 | 10.31 | 14 | 4 | 21 |
| 57 | Q9H3N1 | TMX1 | 2.22 | 9.43 | 4 | 1 | 1 |
| 58 | A6NMY6 | ANXA2P2 | 1.11 | 9.24 | 6 | 2 | 4 |
| 59 | Q9H4I3 | TRABD | 1.61 | 8.54 | 3 | 1 | 1 |
| 60 | Q96F86 | EDC3 | 1.15 | 8.49 | 2 | 1 | 1 |
| 61 | P0DJJ0 | SRGAP2C | 1.69 | 7.35 | 2 | 1 | 1 |
| 62 | Q9UN86 | G3BP2 | 1.15 | 7.16 | 3 | 1 | 2 |
| 63 | P80404 | ABAT | 1.83 | 6.95 | 2 | 1 | 1 |
| 64 | Q06787 | FMR1 | 1.51 | 6.50 | 1 | 1 | 1 |
| 65 | Q8N4C9 | C17orf78 | 1.58 | 6.47 | 4 | 1 | 1 |
| 66 | P10599 | Trx1 | 0.79 | 5.89 | 9 | 1 | 2 |
| 67 | Q14139 | UBE4A | 0.88 | 5.55 | 1 | 1 | 2 |
| 68 | Q7L2H7 | EIF3M | 1.44 | 5.44 | 5 | 1 | 2 |
| 69 | Q6NYC1 | JMJD6 | 1.42 | 5.35 | 3 | 1 | 1 |
| 70 | P43246 | MSH2 | 0.72 | 5.26 | 5 | 5 | 11 |
| 71 | P61964 | WDR5 | 0.87 | 4.98 | 2 | 1 | 1 |
| 72 | P22087 | FBL | 1.41 | 4.92 | 6 | 2 | 3 |
| 73 | Q13823 | GNL2 | 0.83 | 4.50 | 2 | 1 | 1 |
| 74 | Q96Q15 | SMG1 | 1.28 | 3.39 | 0 | 1 | 1 |
| 75 | Q9BSC4 | NOL10 | 0.76 | 3.07 | 1 | 1 | 1 |
| 76 | Q15056 | EIF4H | 0.83 | 2.34 | 4 | 1 | 1 |
| 77 | P12081 | HARS | 0.87 | 2.11 | 3 | 1 | 1 |
| 78 | Q07065 | CKAP4 | 0.89 | 1.97 | 2 | 1 | 1 |
| 79 | P07199 | CENPB | 0.91 | 1.90 | 2 | 1 | 1 |
| 80 | Q9BRS2 | RIOK1 | 0.77 | 1.82 | 2 | 1 | 1 |
| 81 | Q15397 | PUM3 | 0.67 | 1.74 | 1 | 1 | 1 |
| 82 | Q14103 | HNRNPD | 0.73 | 1.54 | 4 | 1 | 6 |

**Table S2.** High-confidence protein candidates identified from in vitro photocrosslinking experiments.

|  | ID | Protein | Abundance Ratio (log2): (Light) / (Heavy) | *P*-Value (−log10): (Light) / (Heavy) | Coverage (%) | Peptides | Peptide spectrum matches |
| --- | --- | --- | --- | --- | --- | --- | --- |
| 1 | Q8N766 | EMC1 | 6.64 | 16.05 | 2 | 2 | 2 |
| 2 | Q8WWN8 | ARAP3 | 6.64 | 16.05 | 1 | 1 | 1 |
| 3 | Q6P1X5 | TAF2 | 6.64 | 16.05 | 1 | 1 | 1 |
| 4 | Q92990 | GLMN | 6.64 | 16.05 | 2 | 1 | 1 |
| 5 | Q8N6H7 | ARFGAP2 | 6.64 | 16.05 | 2 | 1 | 1 |
| 6 | P11717 | IGF2R | 6.64 | 16.05 | 0 | 1 | 1 |
| 7 | Q5T1M5 | FKBP15 | 6.64 | 16.05 | 1 | 1 | 2 |
| 8 | O94763 | URI1 | 6.64 | 16.05 | 2 | 1 | 1 |
| 9 | Q8WXX7 | AUTS2 | 6.64 | 16.05 | 1 | 1 | 1 |
| 10 | O75312 | ZPR1 | 6.64 | 16.05 | 3 | 1 | 1 |
| 11 | Q9GZZ1 | NAA50 | 6.64 | 16.05 | 7 | 1 | 1 |
| 12 | Q13907 | IDI1 | 6.64 | 16.05 | 6 | 1 | 1 |
| 13 | Q9H7Z3 | NRDE2 | 6.64 | 16.05 | 1 | 1 | 1 |
| 14 | P22087 | FBL | 6.64 | 16.05 | 4 | 1 | 1 |
| 15 | Q9Y4W2 | LAS1L | 6.64 | 16.05 | 2 | 1 | 1 |
| 16 | P09211 | GSTP1 | 6.64 | 16.05 | 17 | 2 | 2 |
| 17 | Q9UBN7 | HDAC6 | 6.64 | 16.05 | 1 | 1 | 1 |
| 18 | Q9C0D5 | TANC1 | 6.64 | 16.05 | 1 | 1 | 1 |
| 19 | Q8TD16 | BICD2 | 6.64 | 16.05 | 1 | 1 | 1 |
| 20 | Q5JRX3 | PITRM1 | 6.64 | 16.05 | 1 | 1 | 1 |
| 21 | Q92615 | LARP4B | 6.64 | 16.05 | 1 | 1 | 1 |
| 22 | O76024 | WFS1 | 6.64 | 16.05 | 1 | 1 | 3 |
| 23 | Q7Z3U7 | MON2 | 6.64 | 16.05 | 1 | 1 | 1 |
| 24 | P61964 | WDR5 | 6.64 | 16.05 | 2 | 1 | 1 |
| 25 | Q9H334 | FOXP1 | 6.64 | 16.05 | 1 | 1 | 1 |
| 26 | P60059 | SEC61G | 6.64 | 16.05 | 18 | 1 | 1 |
| 27 | P62847 | RPS24 | 6.64 | 16.05 | 11 | 1 | 1 |
| 28 | P51148 | RAB5C | 6.64 | 16.05 | 6 | 1 | 1 |
| 29 | Q9Y4H2 | IRS2 | 6.64 | 16.05 | 1 | 1 | 1 |
| 30 | O43865 | AHCYL1 | 6.64 | 16.05 | 2 | 1 | 1 |
| 31 | P25787 | PSMA2 | 6.64 | 16.05 | 5 | 1 | 1 |
| 32 | Q92522 | H1FX | 6.64 | 16.05 | 16 | 2 | 3 |
| 33 | Q9BQ69 | MACROD1 | 6.64 | 16.05 | 3 | 1 | 2 |
| 34 | O94819 | KBTBD11 | 6.64 | 16.05 | 2 | 1 | 2 |
| 35 | Q9Y263 | PLAA | 6.64 | 16.05 | 4 | 2 | 3 |
| 36 | Q9ULT8 | HECTD1 | 6.64 | 16.05 | 1 | 2 | 2 |
| 37 | Q8N5N7 | MRPL50 | 6.64 | 16.05 | 7 | 1 | 1 |
| 38 | Q9NWY4 | HPF1 | 6.64 | 16.05 | 3 | 1 | 1 |
| 39 | Q5VTE6 | ANGEL2 | 6.64 | 16.05 | 2 | 1 | 1 |
| 40 | Q9BSJ2 | TUBGCP2 | 6.64 | 16.05 | 1 | 1 | 1 |
| 41 | Q00577 | PURA | 6.64 | 16.05 | 3 | 1 | 1 |
| 42 | Q7Z4H7 | HAUS6 | 6.64 | 16.05 | 1 | 1 | 1 |
| 43 | Q96JB2 | COG3 | 6.64 | 16.05 | 1 | 1 | 1 |
| 44 | P19174 | PLCG1 | 6.64 | 16.05 | 2 | 2 | 3 |
| 45 | Q9Y5A7 | NUB1 | 6.64 | 16.05 | 2 | 1 | 2 |
| 46 | Q96K37 | SLC35E1 | 6.64 | 16.05 | 6 | 1 | 1 |
| 47 | Q08AD1 | CAMSAP2 | 6.64 | 16.05 | 1 | 1 | 2 |
| 48 | O95983 | MBD3 | 6.64 | 16.05 | 3 | 1 | 1 |
| 49 | Q96FV9 | THOC1 | 6.64 | 16.05 | 1 | 1 | 1 |
| 50 | Q8WVM0 | TFB1M | 6.64 | 16.05 | 2 | 1 | 1 |
| 51 | Q9Y485 | DMXL1 | 6.64 | 16.05 | 1 | 1 | 1 |
| 52 | Q9H6S0 | YTHDC2 | 6.64 | 16.05 | 1 | 1 | 1 |
| 53 | Q9H706 | GAREM1 | 6.64 | 16.05 | 1 |  | 1 |
| 54 | Q96MG7 | NSMCE3 | 6.64 | 16.05 | 3 | 1 | 3 |
| 55 | Q9UPS8 | ANKRD26 | 6.64 | 16.05 | 1 | 1 | 1 |
| 56 | Q9P2K3 | RCOR3 | 6.64 | 16.05 | 2 | 1 | 1 |
| 57 | Q9P2R7 | SUCLA2 | 6.64 | 16.05 | 2 | 1 | 1 |
| 58 | Q07864 | POLE | 6.64 | 16.05 | 0 | 1 | 1 |
| 59 | Q9HA65 | TBC1D17 | 6.64 | 16.05 | 2 | 1 | 1 |
| 60 | Q9H1A4 | ANAPC1 | 6.64 | 16.05 | 1 | 1 | 1 |
| 61 | Q03001 | DST | 6.64 | 16.05 | 0 | 1 | 1 |
| 62 | Q9H3H5 | DPAGT1 | 6.64 | 16.05 | 2 | 1 | 1 |
| 63 | P63279 | UBE2I | 5.38 | 16.05 | 5 | 1 | 1 |
| 64 | Q9BRJ2 | MRPL45 | 5.32 | 16.05 | 3 | 1 | 2 |
| 65 | Q9UJX2 | CDC23 | 4.44 | 16.05 | 2 | 1 | 2 |
| 66 | Q9NR56 | MBNL1 | 3.74 | 16.05 | 5 | 2 | 2 |
| 67 | P25054 | APC | 3.46 | 16.05 | 0 | 1 | 1 |
| 68 | Q7L8L6 | FASTKD5 | 3.21 | 16.05 | 2 | 2 | 2 |
| 69 | Q9NYV6 | RRN3 | 3.15 | 16.05 | 2 | 1 | 1 |
| 70 | Q01469 | FABP5 | 3.01 | 16.05 | 7 | 1 | 3 |
| 71 | Q9UPY3 | DICER1 | 2.70 | 16.05 | 1 | 1 | 2 |
| 72 | O75146 | HIP1R | 2.61 | 16.05 | 2 | 2 | 2 |
| 73 | Q8IWF6 | DENND6A | 2.55 | 16.05 | 3 | 2 | 2 |
| 74 | Q14669 | TRIP12 | 2.48 | 16.05 | 2 | 4 | 6 |
| 75 | P10599 | Trx1 | 2.45 | 16.05 | 9 | 1 | 3 |
| 76 | Q15424 | SAFB | 2.42 | 16.05 | 1 | 1 | 1 |
| 77 | Q13671 | RIN1 | 2.34 | 16.05 | 1 | 1 | 1 |
| 78 | Q14697 | GANAB | 1.58 | 14.71 | 1 | 2 | 3 |
| 79 | P48059 | LIMS1 | 2.65 | 14.41 | 6 | 2 | 2 |
| 80 | O95072 | REC8 | 2.58 | 13.87 | 3 | 2 | 2 |
| 81 | P38159 | RBMX | 1.74 | 13.57 | 2 | 1 | 1 |
| 82 | Q9NZJ4 | SACS | 2.22 | 11.91 | 0 | 2 | 2 |
| 83 | O00522 | KRIT1 | 3.25 | 11.33 | 1 | 1 | 1 |
| 84 | P08559 | PDHA1 | 1.18 | 10.53 | 8 | 3 | 10 |
| 85 | Q9H9A5 | CNOT10 | 1.65 | 9.63 | 1 | 1 | 1 |
| 86 | Q9NRL3 | STRN4 | 2.07 | 8.82 | 1 | 1 | 1 |
| 87 | Q5VTB9 | RNF220 | 2.54 | 8.80 | 2 | 1 | 1 |
| 88 | P84243 | H3F3A | 1.03 | 8.74 | 5 | 1 | 7 |
| 89 | Q96I25 | RBM17 | 1.53 | 8.68 | 5 | 2 | 6 |
| 90 | Q9Y496 | KIF3A | 2.06 | 8.55 | 3 | 2 | 4 |
| 91 | P07197 | NEFM | 2.31 | 7.83 | 1 | 1 | 1 |
| 92 | Q99848 | EBNA1BP2 | 1.43 | 7.58 | 3 | 1 | 1 |
| 93 | O14531 | DPYSL4 | 0.92 | 7.54 | 9 | 4 | 9 |
| 94 | O75061 | DNAJC6 | 1.88 | 7.36 | 1 | 1 | 2 |
| 95 | O00217 | NDUFS8 | 2.71 | 7.24 | 5 | 1 | 2 |
| 96 | O15069 | NACAD | 2.42 | 6.59 | 1 | 1 | 1 |
| 97 | Q96BN8 | OTULIN | 2.17 | 6.57 | 2 | 1 | 1 |
| 98 | Q96AG4 | LRRC59 | 0.79 | 6.18 | 16 | 4 | 19 |
| 99 | Q9NYY8 | FASTKD2 | 2.39 | 5.92 | 1 | 1 | 2 |
| 100 | Q2KJY2 | KIF26B | 0.98 | 5.46 | 1 | 2 | 2 |
| 101 | O94822 | LTN1 | 1.75 | 5.30 | 1 | 1 | 1 |
| 102 | Q8ND83 | SLAIN1 | 2.28 | 5.24 | 2 | 1 | 1 |
| 103 | Q14C86 | GAPVD1 | 1.30 | 5.15 | 1 | 1 | 3 |
| 104 | A6NMY6 | ANXA2P2 | 1.63 | 5.11 | 6 | 2 | 3 |
| 105 | P42684 | ABL2 | 1.95 | 5.05 | 2 | 2 | 2 |
| 106 | Q15274 | QPRT | 2.02 | 4.82 | 3 | 1 | 1 |
| 107 | Q9BXC9 | BBS2 | 2.00 | 4.78 | 1 | 1 | 1 |
| 108 | Q8WXH0 | SYNE2 | 2.20 | 4.75 | 1 | 4 | 4 |
| 109 | P18887 | XRCC1 | 0.86 | 4.49 | 4 | 3 | 4 |
| 110 | Q9BRA2 | TXNDC17 | 1.78 | 4.31 | 7 | 1 | 1 |
| 111 | Q7L2H7 | EIF3M | 1.45 | 4.27 | 8 | 2 | 3 |
| 112 | P15104 | GLUL | 1.28 | 4.13 | 3 | 1 | 1 |
| 113 | O14641 | DVL2 | 1.59 | 4.09 | 2 | 1 | 1 |
| 114 | Q7L5D6 | GET4 | 1.97 | 4.07 | 4 | 1 | 1 |
| 115 | P24534 | EEF1B2 | 1.15 | 3.58 | 6 | 1 | 11 |
| 116 | O43390 | HNRNPR | 1.16 | 3.57 | 9 | 5 | 17 |
| 117 | Q63HM1 | AFMID | 1.00 | 3.50 | 3 | 1 | 1 |
| 118 | Q8N6T3 | ARFGAP1 | 1.39 | 3.45 | 8 | 3 | 5 |
| 119 | P17028 | ZNF24 | 1.66 | 3.44 | 3 | 1 | 1 |
| 120 | Q6UX07 | DHRS13 | 1.61 | 3.32 | 3 | 1 | 1 |
| 121 | Q9Y6W5 | WASF2 | 1.52 | 3.26 | 3 | 1 | 1 |
| 122 | Q8IX18 | DHX40 | 1.12 | 2.88 | 1 | 1 | 1 |
| 123 | Q96EE3 | SEH1L | 1.60 | 2.83 | 2 | 1 | 1 |
| 124 | Q99829 | CPNE1 | 1.37 | 2.72 | 1 | 1 | 1 |
| 125 | Q8TBB5 | KLHDC4 | 0.82 | 2.66 | 5 | 2 | 3 |
| 126 | Q9Y2L5 | TRAPPC8 | 1.16 | 2.64 | 1 | 2 | 2 |
| 127 | Q6UW02 | CYP20A1 | 1.34 | 2.49 | 2 | 1 | 1 |
| 128 | Q8NBJ5 | COLGALT1 | 0.62 | 2.48 | 3 | 2 | 2 |
| 129 | Q9HAV4 | XPO5 | 1.22 | 2.48 | 4 | 4 | 4 |
| 130 | P38117 | ETFB | 1.38 | 2.36 | 3 | 1 | 1 |
| 131 | Q9Y2Q9 | MRPS28 | 1.29 | 2.34 | 5 | 1 | 1 |
| 132 | Q99798 | ACO2 | 0.92 | 2.32 | 6 | 4 | 5 |
| 133 | P54577 | YARS | 0.80 | 2.28 | 7 | 4 | 7 |
| 134 | Q15102 | PAFAH1B3 | 0.66 | 2.22 | 7 | 2 | 3 |
| 135 | Q9BW19 | KIFC1 | 1.00 | 2.19 | 5 | 3 | 4 |
| 136 | Q9UJU6 | DBNL | 1.16 | 2.12 | 10 | 3 | 3 |
| 137 | Q6UXV4 | APOOL | 1.35 | 2.12 | 4 | 1 | 1 |
| 138 | Q9H4L4 | SENP3 | 0.67 | 2.09 | 5 | 2 | 2 |
| 139 | Q5JTZ9 | AARS2 | 0.99 | 2.03 | 3 | 3 | 3 |
| 140 | P62491 | RAB11A | 1.31 | 2.00 | 5 | 1 | 1 |
| 141 | Q9UPN4 | CEP131 | 0.95 | 1.99 | 2 | 2 | 3 |
| 142 | Q9Y4P1 | ATG4B | 1.25 | 1.98 | 3 | 1 | 1 |
| 143 | P22570 | FDXR | 1.29 | 1.97 | 2 | 1 | 1 |
| 144 | Q969S9 | GFM2 | 1.25 | 1.88 | 1 | 1 | 1 |
| 145 | Q9UNS1 | TIMELESS | 0.80 | 1.72 | 1 | 1 | 1 |
| 146 | Q4J6C6 | PREPL | 0.84 | 1.70 | 1 | 1 | 2 |
| 147 | Q6PJG6 | BRAT1 | 1.19 | 1.60 | 3 | 2 | 2 |
| 148 | P04040 | CAT | 1.12 | 1.58 | 2 | 1 | 1 |
| 149 | Q08379 | GOLGA2 | 0.63 | 1.54 | 2 | 2 | 3 |
| 150 | O15075 | DCLK1 | 1.07 | 1.46 | 2 | 2 | 2 |
| 151 | Q86VI3 | IQGAP3 | 0.65 | 1.46 | 3 | 5 | 9 |
| 152 | Q15431 | SYCP1 | 1.22 | 1.45 | 1 | 1 | 3 |
| 153 | Q8IZ83 | ALDH16A1 | 1.03 | 1.40 | 1 | 1 | 1 |
| 154 | P62495 | ETF1 | 0.61 | 1.35 | 2 | 1 | 1 |
| 155 | Q9Y6W3 | CAPN7 | 1.03 | 1.34 | 2 | 1 | 1 |
| 156 | Q9Y2S6 | TMA7 | 0.66 | 1.33 | 14 | 1 | 2 |
